# Molecular phylogenetic study of flavonoids in medicinal plants: a case study family Apiaceae

**DOI:** 10.1007/s10265-023-01442-y

**Published:** 2023-02-28

**Authors:** Dalia Youssef, Ranya El-Bakatoushi, Asmaa Elframawy, Laila El-Sadek, Ghada El Badan

**Affiliations:** 1 Biology and Geology Sciences Department, Faculty of Education, University of Alexandria, EgyptAlexandria, El-Shatby, 21526 Egypt; 2 Nucleic Acids Research Department, Genetic Engineering & Biotechnology Research Institute (GEBRI), City for Scientific Research and Technological Applications, Borg El-Arab, Alexandria, 21933 Egypt; 3 Botany and Microbiology Department, Faculty of Science, Alexandria University, Camp Caesar, Alexandria, 21525 Egypt

**Keywords:** APIACEAE, Evolution, Flavonoids, FLS gene, FNS gene

## Abstract

The current study examined the phylogenetic pattern of medicinal species of the family Apiaceae based on flavonoid groups production, as well as the overall mechanism of the key genes involved in flavonol and flavone production. Thirteen species of the family Apiaceae were used, including Eryngium campestre from the subfamily Saniculoideae, as well as *Cuminum cyminum, Carum carvi, Coriandrum sativum, Apium graveolens, Petroselinum crispum, Pimpinella anisum, Anethum graveolens, Foeniculum vulgare, Daucus carota, Ammi majus, Torilis arvensis*, and *Deverra tortuosa* from the subfamily Apioideae. The seeds were cultivated, and the leaves were collected to estimate flavonoids and their groups, physiological factors, transcription levels of flavonol and flavone production-related genes. The phylogenetic relationship between the studied species was established using the L-ribosomal 16 (*rpl16*) chloroplast gene. The results revealed that the studied species were divided into two patterns: six plant species, *E. campestre, C. carvi, C. sativum, P. anisum, An. graveolens,* and *D. carota,* contained low content of flavonoids, while the other seven species had high content. This pattern of flavonoids production coincided with the phylogenetic relationships between the studied species. In contrast, the phylogeny of the flavonol and flavone synthase genes was incompatible with the quantitative production of their products. The study concluded that the increment in the production of flavonol depends on the high expression of chalcone synthase, chalcone isomerase, flavanone 3 hydroxylase, flavonol synthase, the increase of Abscisic acid, sucrose, and phenyl ammonia lyase, while flavone mainly depends on evolution and on the high expression of the flavone synthase gene.

**Supplementary Information:**

The online version contains supplementary material available at 10.1007/s10265-023-01442-y.

## Introduction

The world's use of medical plants is expanding quickly due to the rising demand for herbal medicines, natural health products, and secondary metabolites of medicinal plants (Cole et al. 2007). The Apiaceae family is a significant source of phytochemicals, or compounds with biological action, where phenolic compounds especially phenolic acids and flavonoids are found predominantly (Pandey et al. 2012). Flavonoids have a variety of health advantages, such as antiviral, anticancer, antioxidant, and anti-inflammatory effects. Additionally, they have cardioprotective and neuroprotective properties. These biological processes are dependent on the flavonoid type. As a result, this study examined medicinal plants in the Apiaceae family**,** whose active substances are flavonoids**,** and shed light on the genetic background of flavonoids' biosynthesis by establishing a hypothesis on the relationship between flavonoids' content and phylogenetic relationships. Further, are the key biosynthetic genes of flavonoids also related to family phylogeny**?** This study adds new depth to the family phylogeny by incorporating these active chemicals as a combination of physiological processes and genetics.

Plants have played a unique and comprehensive role in the provision of food, medicine, clothing, and shelter, as well as air quality (Miao et al. 2022). Plants have been used as medicine for more than 5000 years (Brown and Wright 2016), as a source of antibiotics, analgesics, and cardioprotective (Chen et al. 2015). Medicinal plants are important to people, not only as a primary source of medicine but also as phytochemical building blocks for the creation of new drugs (Fabricant and Fransworth, 2001). Recently, it has been recorded that most of the used drugs contain plant extracts due to cultural acceptability, better compatibility and adaptability with the human body, and lower side effects (Oladeji 2016). There has been an increase in interest in the therapeutic potential of medicinal plants, which might be due to their phenolic compounds such as flavonoids, phenolic acids, tannins, coumarins, and lignans (Sharma et al. 2013).

**Fig. 1:**
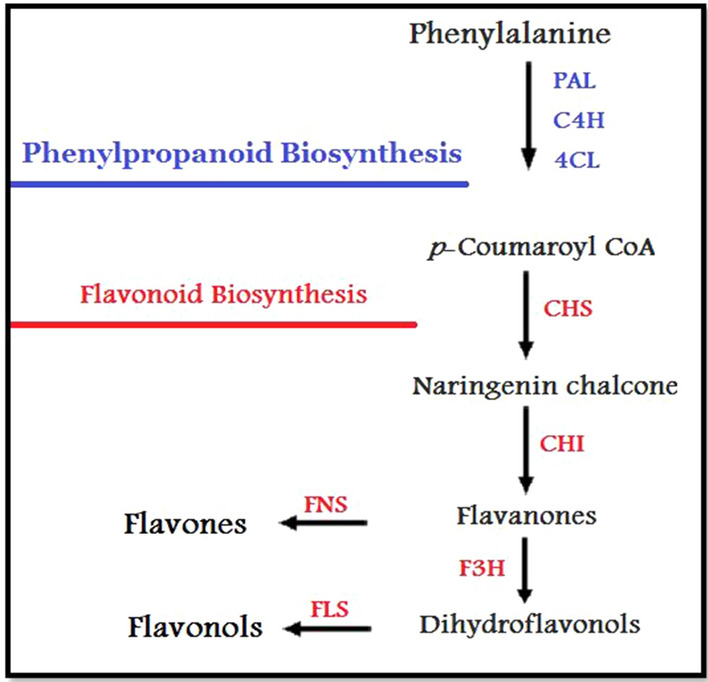
Schematic of a section of the phenylpropanoid pathway leading to the production of major compound groups discussed in the article. Enzyme abbreviations are: *PAL* phenylalanine ammonia-lyase, *C4H* cinnamate 4-hydroxylase, *4CL* 4-coumarate: CoA ligase, *CHS* chalcone synthase, *CHI* chalcone isomerase, *F3H* flavanone 3-hydroxylase, *FNS* flavone synthase, *FLS* flavonol synthase (Davies et al. 2020)

Flavonoids are a widely distributed group of polyphenols that cannot be synthesized by humans or animals (Koes et al. 2005). Chemically, they are based upon the fifteen-carbon skeleton (C6C3C6), consisting of two benzene rings linked via a heterocyclic pyrane ring. According to substitution pattern variations, flavonoids can be divided into a variety of classes such as flavones (e.g., apigenin and luteolin), flavonols (e.g., quercetin, kaempferol, and myricetin), and flavanones (e.g. hesperetin and naringenin) (Dixon et al. 1983). Many nutraceutical, biomedical, medicinal, and cosmetic uses are currently considered indispensable. This is due to their ability to modulate essential cellular enzyme functions, together with their anti-oxidative, anti-inflammatory, anti-mutagenic, and anti-carcinogenic properties (Panche et al. 2016). Flavonols and flavones possess antioxidant and free radical scavenging activity, have significant vitamin C sparing activity and are known for their antiproliferative, anti-angiogenic, and neuro-pharmacological properties (Kim et al. 2006). Flavonols are probably the most relevant flavonoids participating in stress responses, with a wide variety of potent physiological activities (Pollastri and Tattini 2011). Flavones are one of the main subgroups of flavonoids involved in plant-based interactions with various other species, such as microbes and insects. The synthesis of flavonols and flavones depends on the activity of the enzymes flavonol synthase (FLS) and flavone synthase (FNS) in plants (Martens and Mithofer, 2005) (Fig. 1). Possibly the most relevant flavonoids involved in stress responses are flavonols, as they are the most ancient (Pollastri and Tattini 2011).

Chloroplast genomes are more conserved and shorter in length, and they are widely used to determine evolutionary patterns (Jansen et al. 2007) and phylogenetic analysis (Moore et al. 2010). Several studies have successfully used chloroplast sequences to infer relationships at all taxonomic levels, from the deepest level relationships between land plants and Angiosperms (Hilu et al. 2003), through intermediate taxonomic levels of orders and family (Chin et al. 2014; Potter et al. 2007), to relationships among closely related species or populations (Morris et al. 2008; Shaw and Small 2004). Molecular trees based on chloroplast genomes have been used as phylogenetic frames to examine and discuss similarities, and dissimilarities of profiles of secondary metabolites (Wink and Mohammed, 2003). In line with this, Saslis-Lagoudakis et al. (2011) proposed that if a particular category of use exhibits a strong phylogenetic signal, then closely related species will exhibit similar uses. Accordingly, Lukhoba et al. (2006) showed that for the genus *Plectranthus* (Lamiaceae); 62 out of the 300 species are used in some kind of ethnopharmacology preparation. The majority of medicinal species were found within the same large phylogenetic clade, implying that within the genus there is a phylogenetic trend in medicinal properties. Rønsted et al. (2012) also created a phylogenetic hypothesis for the medicinally important plant subfamily Amaryllidaceae based on parsimony and Bayesian analysis of nuclear, plastid, and mitochondrial DNA sequences of over 100 species. They tested the hypothesis that alkaloid diversity and activity in bioassays are significantly correlated with phylogeny. The result of their study confirmed evidence for a significant phylogenetic signal in this trait.

The Apiaceae family, previously known as the Umbel family: Umbellieferae, is derived from the genus Apium, and includes temperate herbaceous annuals (anise, caraway, coriander, cumin, dill, and sweet fennel), biannuals (carrot, parsley, parsnip, and celery), and perennials (angelica, lovage, and bitter fennel). Apiaceae species possess a range of compounds with many biological activities; water-soluble glycosides of monoterpenes, alkyl, and aromatic compounds, as well as, cellulose, lignin, wax esters, pectin, phospholipids, flavonoids, carotenoids, and terpenoids (Jia et al. 2015). Consequently, some plants from this family are used as home-based remedies to treat various digestive, endocrine, reproductive, and respiratory diseases (Aćimović et al. 2015). Moreover, they have the potential of antioxidant activity, digestive stimulant action, anti-inflammatory, antimutagenic, anticarcinogenic, and anti-hypertensive activities (Eidi et al. 2009; Lampe 2003).

Egypt's flora has a wealth of valuable medicinal plant species. The objectives of the present study were: (1) to elucidate the overall mechanism of flavonoids production in the studied plant species of the family Apiaceae; (2) to generate a supported Apiaceae phylogenetic hypothesis based on chloroplast ribosomal protein 16 (*rpl16*) DNA evidence and test for a relationship between phylogenetic and flavonoids diversity in the Apiaceae family. (3) Characterization of key genes of flavonol and flavone subgroups, *FLS* and *FNS* genes, and their relationship to the family's phylogeny.

## Material and methods

Thirteen species of medicinal plants of the family Apiaceae were used; nine of which were cultivated species: *Cuminum cyminum* L., *Carum carvi* L., *Coriandrum sativum* L., *Apium graveolens* L., *Petroselinum crispum* H., *Pimpinella anisum* L., *Anethum graveolens* L., and *Foeniculum vulgare* M.; and four wild species (fresh from field) *Ammi majus* L., *Torilis arvensis* (Huds.) Link., *Deverra tortuosa* DC., and *Eryngium campestre* L. All species are from the subfamily Apioideae, except one species, *Eryngium campestre*, which is from the subfamily Saniculoideae. The cultivated plants of the family Apiaceae were kindly supplied by the Agricultural Research Center, Itay Al Barud, Egypt. Meanwhile, wild species were collected from different sites in Alexandria City; *Ammi* sp. and *Deverra* sp. were collected from Borg El-Arab El-Kadeem. *Eryngium* sp*.* and *Torilis* sp. were collected from El-Hammam and El Shohada Square Road, Alexandria, respectively, and were identified by Täckholm (1974) and Boulos (1999).

### Seed germination

Seeds were surface scratched with sandpaper to break dormancy, sterilized by soaking for two minutes in 10% (v/v) sodium hypochlorite, washed several times with distilled water, and then soaked for 24 h in distilled water to stimulate germination. The seeds were transferred to plastic pots filled with a fixed amount of previously acid-washed quartz sand. Pots were placed under natural environmental conditions and were irrigated for 28 days using a half-strength modified Hoagland nutrient solution (Epstein 1972). The leaves of seedlings were collected and prepared for physiological analysis and molecular studies (3 replicates).

### Estimation of total flavonoids content

The content of flavonoids in the examined plant extracts was determined using the spectrophotometric method (Quettier et al. 2000). The sample contained 1 ml of extract and 1 ml of a 2% aluminum trichloride (AlCl3) solution. The samples were incubated for an hour at room temperature. The absorbance was determined using a spectrophotometer at a maximum λ of 415 nm. The samples were prepared in triplicate for each analysis and the mean value of absorbance was obtained. The same procedure was repeated for the standard solution of Rutin (with concentrations ranging from 10 to 100 µg ml^−1^) for the calibration line construction. The content of flavonoids in extracts was expressed in terms of rutin equivalent (µg per Rug fresh weight).

### Flavone and flavonol content estimation

The content of both Flavonol and flavone combined was measured according to Bonvehi et al. (1994). An aliquot (2 ml) of the plant extract and 1 ml of 5% aluminum trichloride in methanol (w/v) were mixed. The mixture was left for 30 min and the absorbance was 425 nm. The same procedure was repeated for the standard solution of quercetin (with concentrations ranging from 10 to 70 µg ml^−1^) and the calibration line was constructed. Regarding flavonol content only, it was determined according to Yermakov et al. 1987. The procedure was carried out with 2 ml of plant extract mixed with 2 ml (20 g l^−1^) aluminum trichloride and 6 ml (50 g l^−1^) sodium acetate. The absorption at 440 nm was measured after incubation for 2.5 h at 20 °C. All determinations were carried out in duplicates, and the content of flavonols was calculated in quercetin equivalents. Through deducting the content of flavonols only (440 nm absorbance) from the content of both flavonols and flavones (425 nm absorbance), the flavone content was estimated in the samples.

### Determination of physiological factors affecting flavonoids production

#### Estimation of Invertase Hydrolyzate Sugars (IHS)

Reducing sugars (RS) were determined by the method described by Dubois et al. (1956). To 2 ml of the clarified extract, 1 ml of 5% phenol followed by 5 ml of concentrated H2SO4 were added rapidly. The tubes were allowed to stand for 10 min, and the absorbance was recorded at 490 nm. Sucrose as a non-reducing sugar was determined enzymatically. At pH 4.6, sucrose is hydrolyzed by invertase into glucose and fructose. To an aliquot of the water-soluble extract (2 m1), 1 ml of 0.1% invertase (Merck) was added. The mixture was incubated in a water bath at 35 °C for 30 min. The hydrolyzate was made up to a known volume (10 ml) and the total reducing value (TRV) was determined by absorbance at 490 nm. The invertase hydrolyzate fraction (IHS) was calculated by subtracting RS from the TRV value. A calibrated curve using pure glucose was made from which the amount of sugar was calculated as mg g^−1^ fresh wt.

#### Determination of L-phenylalanine ammonia-lyase activity (PAL, EC 4.3.1.5)

The extraction was made following the procedure described by Morello et al*.* (2005). Approximately 300 mg of fresh plant leaves were homogenized in cooled potassium phosphate buffer (0.05 M, pH 6.6) with 0.2 g of Triton X-100 and centrifuged at 4 °C for 15 min at 6000 rpm. l-Phenylalanine ammonia lyase activity was assayed using the Kagalea et al. (2004) method. The assay mixture contained the following components in a final volume of 3 ml: 0.5 ml of 16 mM L-phenylalanine, 1.5 ml of 50 mM Tris–HCl buffer (pH 8.9), 0.8 ml of 3.6 M NaCI, and 0.2 ml of the crude enzyme extract. Incubation was performed at 37 °C for 1 h and the reaction was stopped by the addition of 0.5 ml of 35% (w/w) of trifluoroacetic acid (TFA). The mixtures were centrifuged for 5 min at 5000 rpm to pellet the denatured protein. The cinnamic acid yield in the supernatant (from hydrolysis of phenyl alanine by the enzyme) was estimated by measuring the absorbance at 290 nm using a spectrophotometer (Unico 2100, USA).

#### Determination of ABA content

Fresh weight samples (0.4 g) were extracted after the addition of internal standards with 500 µl of solvent extract (methanol: isopropanol: glacial acetic acid, 20: 79: 1, v/v) for 24 h at 4 °C. The samples were centrifuged at 4 °C for 15 min at 10,000 rpm (Muller and Munné-Bosch, 2011). The endogenous abscisic acid (ABA) level was determined in the aqueous phase by the method described by Kelen et al. (2004). The high-performance liquid chromatography (HPLC) system consisted of an Agilent C181200 series HPLC apparatus (Agilent Technologies, Santa Clara, CA, USA), including a high- pressure binary-gradient solvent-delivery pump, and DAD (diode-array detector). An Agilent C18 column (250 mm, 5 µm) was used, and the temperature was maintained at a constant 25 °C. Mobile phases used were acetonitrile–water (30: 70%; v/v) containing 30 mM phosphoric acid, at pH 4.0 (an isocratic elution), with a flow rate of 0.8 ml min^−1^. The detection wavelength was 265 nm. An injection volume of 100 µl was used for each analysis. Chromatography was conducted separately to determine the retention time for each corresponding sample. To quantify the ABA content, a stock solution of ABA was diluted by methanol grade HPLC to obtain known concentrations of ABA (20–100 µg ml^−1^). ABA solutions were injected into the HPLC system and the equation, correlating peak area to ABA concentration, was formulated. The calibration equation of ABA was obtained by plotting HPLC peak areas (y) versus the concentration of calibrators (x), as follows: y = 81.26x + 84.86 (*R*^*2*^ = 0.999).

### Real-time PCR (RT-PCR) expression analysis of flavone and flavonol biosynthesis key genes

RNA was extracted by using the EZ-10 -Spin Column Total RNA Minipreps Super Kit (BIO BASIC, Canada). Reverse transcription reactions were performed using Reverse Aid TM M-MLV reverse transcriptase (Invitrogen). Each 20 µl of reverse-transcription mixture contained 3 µl total RNA, 2 µl reverse transcriptase buffer, 2 µl dNTPs, 3 µl oligo (dT)-primer, and 0.3 µl (I U) reverse transcriptase. The samples were incubated at 42 °C for 60 min, and then at 72 °C for 10 min to inactivate the reverse transcriptase. This method was modified from Carginale et al. (2004). cDNA samples were stored at − 20 °C for subsequent PCR reactions.

The primers used in this study for the amplification of specific genes related to the flavone and flavonol pathway were designed according to genes of chalcone synthase (*CHS*) (AJ006779.1, KC820130.1, KP726914.1, AF177944.1), chalcone isomerase (*CHI*) (KP726914.1, KM359964.1), Flavanone 3β- hydroxylase (*F3H*) (DQ683351.1, AF184270.1, KC820134.1, AY230248.1), *FLS* (AY230249.2, XM_017395104.1), *FNS* (AY230247.1, DQ683352.1, DQ683350.1, MF197308.1, AY817676.1, AY817675.1), and Actin gene (AB181991), using the Jalview program (Waterhouse et al. 2009) and confirmed by Primer-BLAST (https://www.ncbi.nlm.nih.gov/tools/primer-blast/index.cgi).These primers were manufactured by Clinilab Biotechnology Company (Table 1).

**Table 1:** Sequences of primers used in real time PCR

Gene	Primer Sequence	
CHS	F	5-ACTGGAACTCCTTCTTCTGGA-3
	R	5-AACCCGAAAAGAACACCCCA-3
CHI	F	5- GTTACAGGTCCCTTTGAGA-3
	R	5- GCTTTCCAATGAGCAACAC-3
F3H	F	5-GGTGAAGCGGTGCAGGATTG-3
	R	5-ATCAGGTIGTGGGCACTTGGG-3
FNS	F	5-AATTACTATCCCACATGCCC-3
	R	5-CCAGCTTGGCCTTCTCTTCT-3
FLS	F	5- AGGTCGGAAAACGAACAACC-3
	R	5- CTTCGTTAGGTACAAGAATGG-3
Actin	F	5-TTGTGCTCGACTCTGGTGAT-3
	R	5-GCTCATAATCAAGGGCCACG-3

F = forward, R = reverse

The relative expression levels of the target genes *CHS, CHI, F3H, FLS,* and *FNS* in plant species under study were determined on the basis of normalization with the constitutive gene (β- actin gene) using the specific primers for amplification of both target and reference genes as shown in Table 1. The used PCR reaction is the following protocol: 95 °C for 2 min (1 cycle), 95 °C for 5 s., 60 °C for 10 s., 95 °C for 20 s. (40 cycles), and 72 °C for 2 min. Expression of genes was determined by quantitative RT-PCR; CFX Connect Real-Time PCR Detection System (www.bio-rad.com/en-eg/product/cfx-connect-real-time-per-detection system ID = LN5TFG15) with the SYBR Green reagent (SensiFASTTM SYBR® No-ROX Kit).

The expression level of target genes was normalized using the in-run beta actin gene as an internal control and relative transcript levels (RTL) were calculated as follows: RTL = 2^− ΔCt^, where ΔCt = CT (target gene)—CT (constitutive control gene) (Livak and Schmittgen 2001).

### Characterization of chloroplast ribosomal L16 (rpl16) intron region and key genes for flavonols and flavone production (FLS and FNS)

Plant genomic DNA from fresh leaves was extracted by CTAB DNA Extraction Protocol (Cullings 1992). Ribosomal protein L16 intron regions were amplified by PCR using universal primer pairs, F- 5-ATGTTGTTTACGGAATCTGG-3, R- 5-ATGCTTAGTGTGCGACTCGTT-3 (Nicolas and Plunkett 2009). While the *FLS* and *FNS* genes were amplified by using primer pairs used in RT-PCR, which are shown in Table 1, The reaction mixture of PCR was carried out in a total volume of 50 µl containing 25 µl COSMO PCR Master Mix (Willowfort Corporation, Birmingham, UK), 2 µl of 10 pmol forward and reverse primers, 2 µl DNA, and 10 µl of sterile water. PCR amplification was carried out with one cycle of an initial 3 min denaturation at 95 °C, 35 amplification cycles were then performed as follows: 1 min at 95 °C for denaturation, 1 min at 57 °C for annealing and 1 min at 72 °C for elongation. The reaction was then incubated at 72 °C for 5 min for a final extension, followed by a 4 °C hold. The amplification of *rpl16*, *FLS* and *FNS* genes was carried out by the Applied Biosystems Verti Thermal Cycler (https://www.thermofisher.com/eg/en/home/lifescience/pcr/thermal cyclers realtime-instruments/thermal-cyclers/veriti-thermal-cycler.html). Amplified PCR products were separated in a 1.5% agarose gel, 5 µl was loaded per gel slot. The gel was stained with an ethidium bromide solution. The size of each band was estimated by using a 100 bp DNA ladder. Clear amplified bands of PCR products were cut from the gel and subjected to purification and sequencing. An EZ-10 Spin Column DNA Gel Extraction Minipreps kit (BIO BASIC INC) was used to purify the amplified products (bands = genes) for sequencing. The DNA sequences for the respective genes were performed by Macrogene Company (Korea). Sequences of the studied genes were aligned with retrieved sequences from NCBI using ClustalX (Higgins and Sharpe, 1988). These retrieved sequences were used as outgroups: *rpl16* gene from Aralia spinosa (AF094458.1) and *Pittosporum dallii* (AF094469.1); *FLS* from *Lonicera japonica* (JQ627647.1) and *Lactuca sativa* (AB359897.1); *FNS* from *Lonicera japonica* (JX068612.1) and *Dahlia pinnata* (AB769842.1). Neighbour- joining and Maximum likelihood (MP) were employed in the phylogenetic analysis of the datasets, using MEGA-X (Swofford 2001). One hundred replicates were run under the TBR branch-swapping algorithm, saving no more than 1000 trees per replicate. Clade support was estimated using MEGA-X to calculate bootstrap values (Felsenstein 1985).

### Statistical analysis

A one-way ANOVA with the LSD at *p* < 0.05 and Duncan's New Multiple Range Test at the 5% level were used to analyze the data. Also, Pearson correlation tests were computed. For all statistical procedures, a significant level of *p* < 0.05 was employed using SPSS version 13 software, according to Sokal and Rohlf's technique (1995).

## Results

### Flavonoids content

**Fig. 2:**
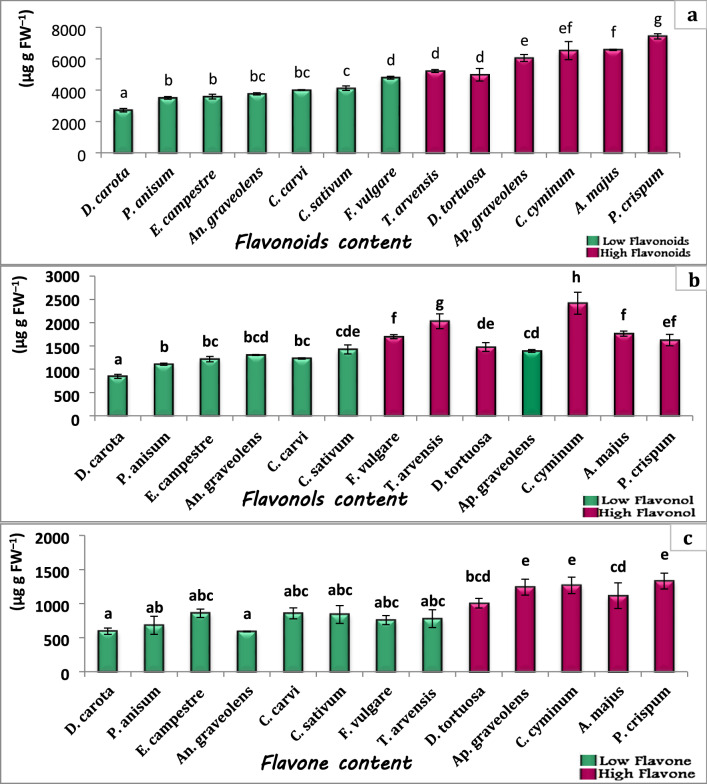
Flavonoids contents **a** and their subgroups flavonol **b** and flavone **c** in the studied plant species. The values reported in the Figure are means (*n* = 3) ± standard deviation, different letters on the bars are significantly different as evaluated by Duncan’s New Multiple Range Test

Total flavonoids showed a wide range of concentrations from 2720 to 7430 µg g fresh weight (FW) ^−1^ in the studied plant species. The highest concentration of flavonoids was recorded in *P. crispum* followed by 6571 in *A. majus*. Compared with the mean value of flavonoids (4866 µg g^−1^) there were seven plant species with values lower than the mean, these included; *D. carota, P. anisum, E. campestre, An. graveolens, C. carvi, C. sativum* and *F. vulgare*. Meanwhile, the other six species showed flavonoids values higher than the mean. These included; *T. arvensis, D. tortuosa, Ap. graveolens, C. cyminum, A. majus* and *P. crispum.* According to flavonol content, the lowest value of flavonol content (845 µg g FW^−1^) was recorded in *D. carota* while the highest concentration (2420 µg g FW^−1^) was observed in *C. cyminum*. Consistently, the flavonol pattern in the studied species was like flavonoids, except that *F. vulgare* has higher content than the mean. Given flavone content, it showed values lower than flavonol in the studied species, ranged from 590 µg g FW^−1^ in *An. graveolens* to 1330 µg g FW^−1^ in *C. cyminum* and *P. crispum*. It was noteworthy that three plant species, *A. majus, C. cyminum* and *P. crispum* have a high concentration of both flavonol and flavone content. While the two species, *D. tortuosa* and *Ap. graveolens*, have high flavone content. *F. vulgare* and *T. arvensis* showed high flavonol content only. Meanwhile, the remaining six plant species had low flavonol and flavone content (Fig. 2a, b, c).

### Physiological factors related to flavonoids production

**Fig. 3:**
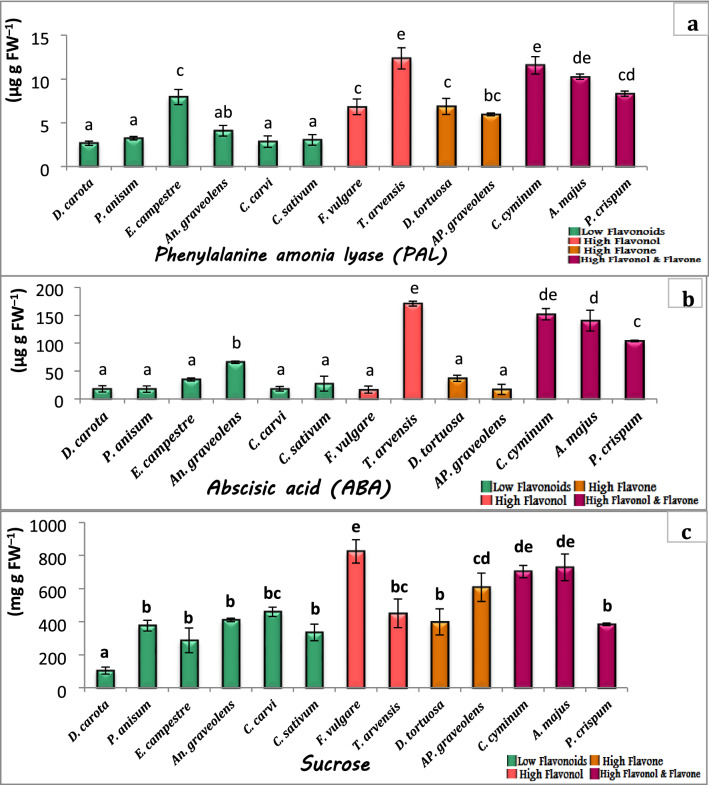
Physiological factors related to flavonoids production. **a** PAL activity. **b** ABA content. **c** Sucrose content, in the studied plant species. The values reported in the figure are means (*n* = 3) ± standard deviation, different letters on the bars are significantly different as evaluated by Duncan’s New Multiple Range Test

The key enzyme for the metabolism of phenols is L-phenylalanine ammonia lyase (PAL). The highest activity of PAL was 12.5 µg g FW^−1^ recorded in *T. arvensis* (high in flavonol content), while *C. carvi* showed the lowest activity (2.8 µg g FW^−1^). Consistently to PAL, the plant hormone ABA showed the highest concentrations in *T. arvensis* (high in flavonol content) followed by the three plant species *C. cyminum*, *A. majus* and *P. crispum* while the rest of the species showed lower content of ABA. Regarding sucrose sugar content, the variation between plant species was not clear. The highest content (825 mg g FW^−1^) was recorded in *F. vulgare* which have a high content in flavonol, while the lower content (105 mg g FW^−1^) was recorded in *D. carota* (low in flavonoids) (Fig. 3a, b, c).

### Gene expression analysis of the flavonol–flavone pathway

**Fig. 4:**
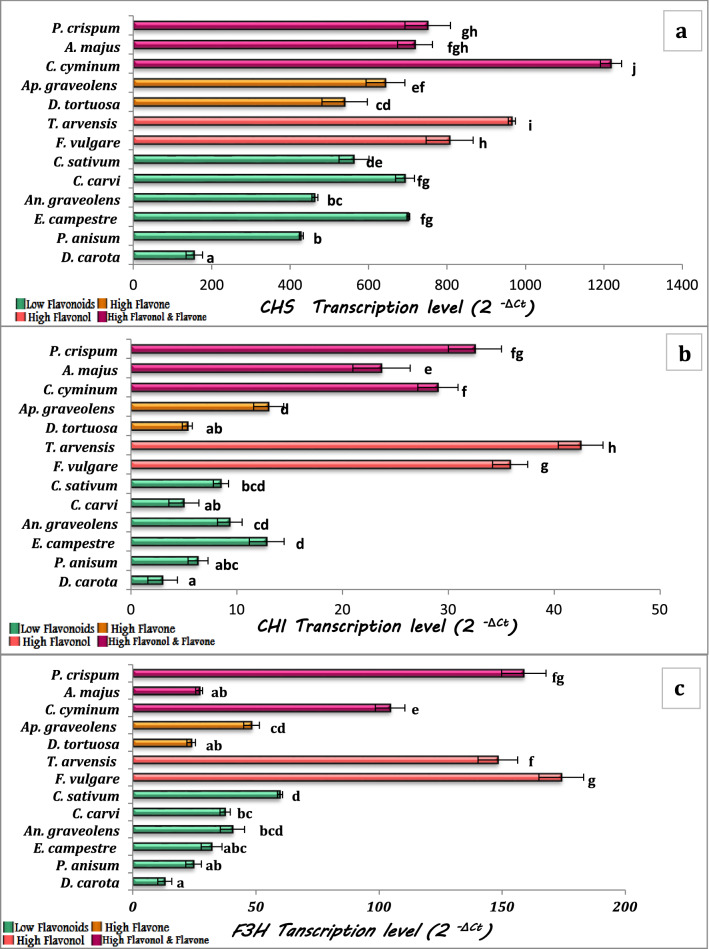
Transcription levels of *CHS* (**a**), *CHI* (**b**) and *F3H* (**c**) in the studied plant species. The values reported in the figure are means (*n* = 3) ± standard deviation, different letters on the bars are significantly different as evaluated by Duncan’s New Multiple Range Test

**Fig. 5:**
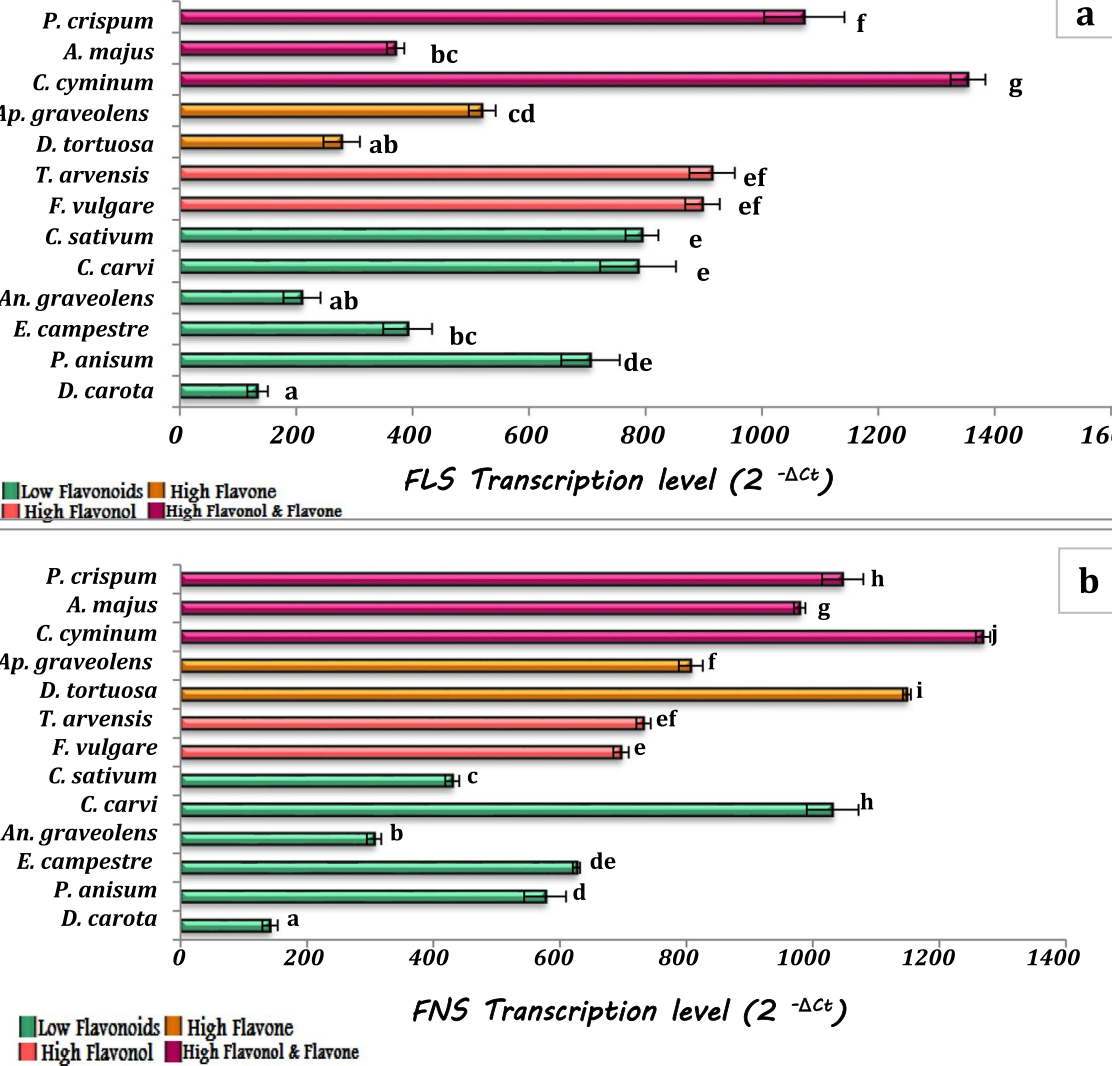
Changes in transcription levels of *FLS* (**a**) and *FNS* (**b**) in the studied plant species. The values reported in the figure are means (*n* = 3) ± standard deviation, different letters on the bars are significantly different as evaluated by Duncan’s New Multiple Range Test

In this study, the expression pattern of genes related to flavone and flavonol biosynthesis in plant leaves was investigated. Chalcone synthase (CHS) is the first enzyme in the flavonoid pathway. The transcription levels of *CHS* showed high levels in *C. cyminum* and low transcription levels were observed in *D. carota* and *P. anisum*. While the second enzyme in the flavonoids pathway CHI, showed higher transcription levels in high flavonol content species; *T. arvensis* and *F. vulgare*, and species of high flavonol and flavone content (*P. crispum, C. cyminum,* and *A. majus*), and showed low transcription levels in the rest of the species. Except for *A. majus*, *F3H* and *FLS* genes were found at high levels in the same species with high flavonol content only and species with both high flavonol and flavone content. On the other hand, the transcription levels of *FNS*, a key enzyme in flavone synthesis, did not show a clear variation between species. The high levels were recorded in *C. cyminum* and *D. tortuosa,* while the low transcription levels were observed in *D. carota* and *An. graveolens.* Overall, the transcription levels of the studied genes revealed that the four plant species, *C. cyminum*, *P. crispum* (high in both flavonol and flavone content), *T. arvensis,* and *F. vulgare* (high only in flavonol content), showed the highest transcription levels of genes for flavonol biosynthesis (*CHS*, *CHI*, *F3H,* and *FLS*). Regarding flavone synthesis, *C. cyminum*, *P. crispum,* and *D. tortuosa* (high in flavone content only) showed the highest transcription levels of flavone synthesis genes *CHS*, *CHI,* and *FNS*. At the same time, it is obvious that *D. carota* showed the lowest level of transcription of all studied genes (*CHS*, *CHI*, *F3H*, *FLS,* and *FNS*) related to the flavonol-flavone pathway (Figs. 4 and 5).

### Analysis of principal components and clustering

**Fig. 6:**
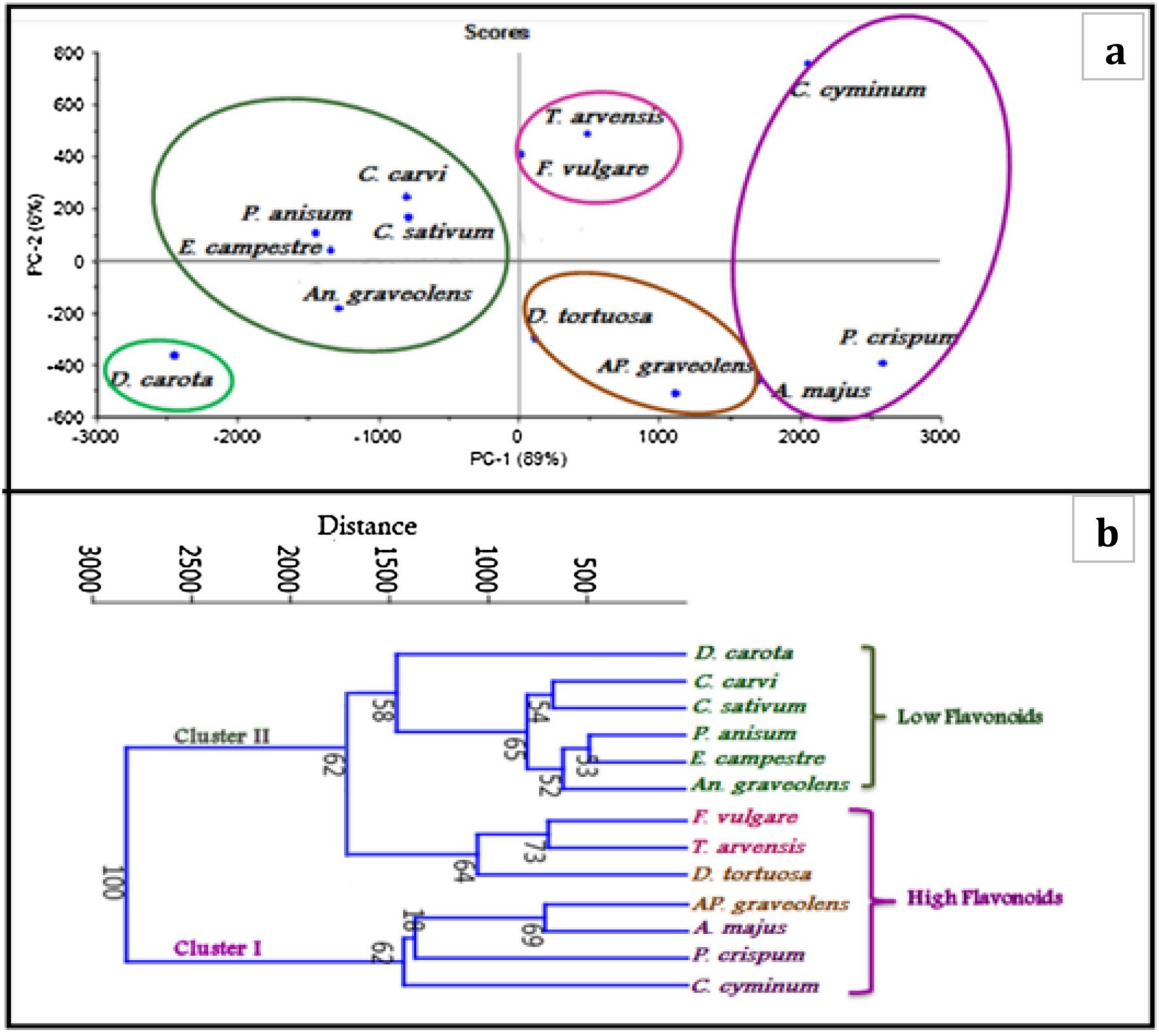
**a** Principal component analysis (PCA) of the studied plant species, **b**: Denderogram of plant species based on phytochemical, physiological factors and genes expression using Euclidean coefficient. Bootstrap values are presented at each clade

The principal component analysis (PCA) was applied to analyse the plant species using 11 variables, including flavonoids content (total-subgroups), physiological factors (sucrose, PAL, and ABA), and gene transcription level (*CHS*, *CHI*, *F3H*, *FLS*, and *FNS*). The first two components, PCA1 and PCA2, represented 89.92% and 6.20% of the total variation, respectively, comprising together 96.12% of the difference between the data. The PCA2 clearly separated the plant species into two clusters, which corresponded to groups classified according to the mean flavonoids content. Cluster1 included all species of low flavonoids content; *P. anisum*, *E. campestre*, *An. graveolens*, *C. carvi*, *C. sativum* and *D. carota*. The second cluster included all species with high flavonoids content, which were divided into three groups: the first contained two species with high flavonol content (*T. arvensis* and *F. vulgare*); the second contained species with high flavone content (*D. tortuosa* and *Ap. graveolens*); and the third group included *C. cyminum*, *P. crispum*, and *A. majus*, which had high flavonol and flavone content. The dendrogram was obtained by applying the UPGMA method using the Manhattan coefficient. Cooperatively with PCA investigations, the cluster analysis indicated the discrimination of plant species into two clusters (1 and 2) at a 3000 coefficient. Cluster 1 included three species with high flavonol and flavone content: *C. cyminum*, *P. crispum*, and *A. majus*, as well as *Ap. graveolens* with high flavone content at 1500 coefficient. The second one comprised two clades; clade1 at 1100 coefficient with 64% bootstrap, contained *F. vulgare*, *T. arvensis* (high in flavonol) and *D. tortuosa* (high in flavone). Clade 2 separated *D. carota* in one branch at 1500 coefficient (58% bootstrap) and the rest of the species with low flavonoids content in one group at 800 coefficient: *P. anisum*, *E. campestre*, *An. graveolens*, *C. carvi*, and *C. sativum* (Fig. 6 a, b).

### Phylogenetic study

**Fig. 7:**
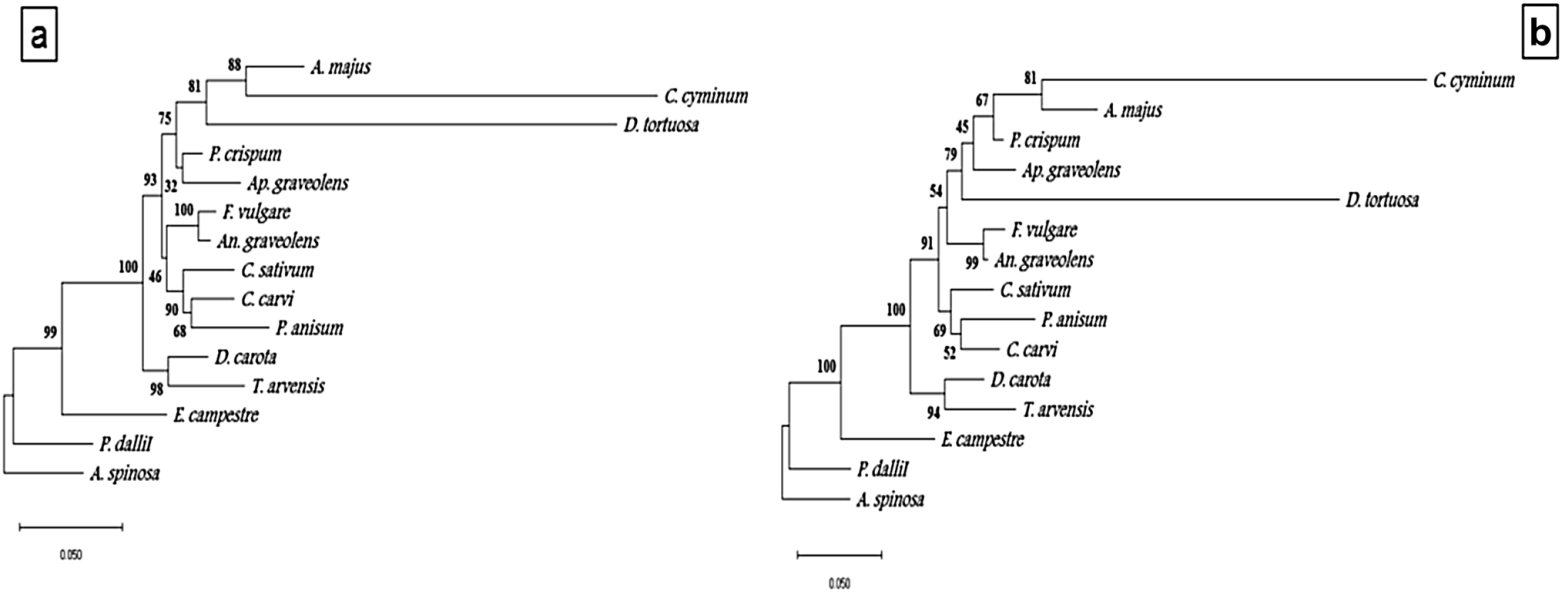
Phenogram of the studied plant species based on cpDNA *rpl16* sequences by different methods **a**: Neighbour-joining **b**: Maximum likelihood

The sequence of the *rpl16* gene intron region (with accession numbers of MW036658 to MW036670) in plant species showed that DNA length ranged from 838 bp in *A. majus* to 940 in *P. anisum*. The A + T fragment content ranged from 67.06 to 69.4% and was two-fold higher than the G + C content, which ranged from 30 to 32% (Figs. S1 and S2, and Table S1). Phylogenetic analyses based on the sequenced intron of *rpl16* gene regions were applied to the studied plant species. The phenogram resulted from using the neighbor-joining method revealed the separation of *E. campestre* species (Subfamily Saniculoideae). The other species of sub-family Apioideae formed a monophyletic group and were separated into two clusters, which are strongly supported by 100% bootstrap (BS). Cluster 1 included two plant species, *D. carota* and *T. arvensis,* from low and high flavonoids groups respectively. Cluster 2 comprises two clades supported with 93% BS. *P. anisum*, *C. carvi*, *C. sativum*, *An. graveolens*, and *F. vulgare* were the five species with the lowest flavonoids content in Clade 1. Clade 2, supported with 75% BS comprises the two species of high flavone content (*Ap. graveolens* and *D. tortuosa*) and three species of high flavonol and flavone (*P. crispum*, *C. cyminum* and *A. majus*). The same result was obtained by the maximum likelihood phenogram (Fig. 7a, b**).**

### Characterization of flavonol synthase (FLS) and flavone synthase (FNS) genes

**Fig. 8:**
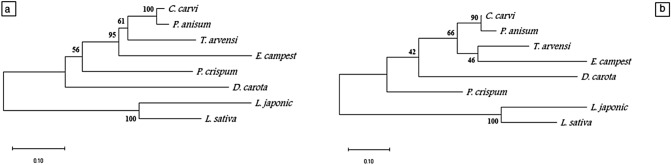
Phenogram of the studied plant species based on *FLS* sequences by different methods. **a:** Neighbour-joining, **b**: Maximum likelihood

**Fig. 9:**
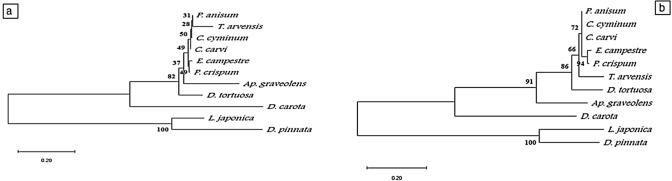
Phenogram of the studied plant species based on *FNS* sequences by different methods. **a** Neighbour-joining, **b**: Maximum likelihood

Six of the 13 studied plant species of the family Apiaceae were used to amplify the *FLS* gene. The amplification yielded a fragment with approximately 800 bp (with accession numbers MW036671 to MW036676). Alignment of all six sequences resulted in a matrix of 940 positions, of which 361 characters were conserved, 507 variables (53%), and 59 (62%) CpG sites (Figs. S3 and S4, and Table S2). The phylogeny analyses based on FLS gene region sequences were applied to the studied plant species. The phenogram resulted from using the neighbour -joining method revealed the separation of *D. carota* from the other studied species. The remaining five plant species of Apiaceae were divided into; one branch contained *P. crispum* of high flavonol and flavone content, and one clade with a high bootstrap value (95%) contained the three species of group 1 of low flavonoids (*P. anisum*, *C. carvi,* and *E. campestre*) and one species (*T. arvensis*) of high flavonols content. The maximum likelihood phenogram was identical to the one produced by the neighbour-joining method, except for a slight difference in bootstrap values (Fig. 8a,b). Nine of the 13 studied plant species were used to amplify *FNS*. The amplification yielded a fragment with an average size of 600 bp (with accessions NO. MW036677 to MW036685). Alignment of all nine sequences resulted in a matrix of 853 positions, of which 273 characters were conserved (32%), 386 variables (45%), and 27 (31%) CpG sites or CG sites (Figs. S5 and S6, and Table S3). The phenogram resulted by using neighbour-joining method revealed the separation of *D. carota*. The remaining eight plant species of Apiaceae were divided at 82% bootstrap into a branch of *D. tortuosa* of high flavone content and one clade including: *Ap. graveolens* (high in flavone); and *E. campestre*, *P. anisum*, and *C. carvi* (low in flavone content); *P. crispum*, and *C. cyminum* (high in flavonol and flavone content) and the high flavonol species *T. arvensis*. The maximum likelihood phenogram, was similar to those resulting from the neighbour-joining method, with high bootstrap values. The only difference between the two methods is the separation of *T. arvensis* at 66% bootstrap from *E. campestre*, *P. anisum*, *C. carvi*, *P. crispum,* and *C. cyminum (*Figs. 8 and 9a, b).

## Discussion

Many studies are being conducted to address the question of how to discover plants that contain drugs. Ethnomedicinally important plants are a good starting point to focus on yielding new drugs. Flavonoids characterize a large class of secondary plant metabolites with low-weight phenolic compounds. In 33 categories of plant uses in South Africa, Yessoufou et al. (2014) evaluated the phylogenetic signal and concluded that the taxa that are evolutionarily closely related have significantly more uses than those that are evolutionarily isolated. They have also supported the importance of phylogeny in bioscreening medicinal flora for the discovery of alternative medicines. In plants, flavonoids play an important role in UV protection, defense against pathogens and ultraviolet radiation, microorganism signaling, controlling the transport of auxin and pigmentation (Li et al. 2016; Wei et al. 2015; Winkel-Shirley 2001). The current study showed that six plant species (*D. carota*, *P. anisum*, *E. campestre*, *An. graveolens, C. carvi, and C. sativum*) have lower flavonoid content. While the three species, *P. crispum*, *A. majus,* and *C. cyminum,* have a high content of flavonol and flavone, *F. vulgare* and *T. arvensis* were high in flavonol content, while *Ap. graveolens* and *D. tortuosa* were high in flavone. This investigation of plant species that have high and low flavonoid content coincided with the recent study by Shawky and Abou El Kheir (2018) on some Apiaceae plant species from Egypt. They found that high flavonoid content species; *C. cyminum, P. crispum*, *Ap. graveolens*, *F. vulgare,* and *A. majus*, were clustered together, while plant species with low flavonoid content, such as *P. anisum*, *C. sativum*, *C. carvi,* and *An. graveolens,* were grouped together. In the current study, the highest values of flavonols were recorded in *C. cyminum, P. crispum*, and *F. vulgare* species, while the highest values of flavone were demonstrated in *Ap. graveolens*, *P. crispum,* and *C. cyminum*. This result coincides with Orhan et al. (2016) who studied the antioxidant activity of certain species of Apiaceae (*P. crispum, Ap. graveolens*, *P. anisum*, *F. vulgare,* and *C. sativum*) and showed that *P. crispum* has the highest value of flavonoids. However, Shah et al. (2014) compared flavonoids content of *C. carvi*, *C. sativum, An. graveolens,* and *C. cyminum* and found that *C. carvi* has the highest flavonoid content, which is contrary to the present study.

Concerning flavonol content, it was found that it was significantly correlated with the activity of the PAL enzyme and the plant ABA (PAL and ABA (*r* = 0.833)), sucrose, and transcription level of the related studied genes, *CHS*, *CHI* and *F3H*. The plant hormone ABA activates the first committed enzyme in the general phenylpropanoid pathway, PAL (Di et al. 2017). As observed in the roots of the maize cultivar Zhengdan 958 (Tian and Li,2018) and *Dracocephalum moldavica* (Khaleghnezhad, et al. 2019), ABA causes a significant increase in total flavonoid content through increasing PAL activity. Regarding sucrose content, the present study showed a high correlation with flavonoids and flavonols in the studied species. This result agrees with Solfanelli et al. (2006), who revealed that flavonoids and anthocyanin biosynthetic pathways are strongly up-regulated following sucrose treatment. They also showed that among different sugar treatments (sucrose, glucose, and fructose), sucrose alone caused induction in the genes coding for flavonoid and anthocyanin biosynthetic enzymes in *Arabidopsis thaliana*.

The biosynthesis of flavonoids in plants is closely related to the expression of genes encoding their biosynthetic enzymes. In the current study, flavonol production in *F. vulgare*, *T. arvensis*, *C. cyminum,* and *P. crispum* was significantly increased by the increase in transcription levels of *CHS*, *CHI,* and *F3H* genes. In the meantime, the lowest transcription levels of flavonoids pathway genes (*CHS*, *CHI*, *F3H*, *FLS*, and *FNS*) were detected in *D. carota,* which has a low content of flavonoids (flavonols and flavones). The increase in the transcription level of flavonol production genes is reported in different studies like, Xu et al. (2014), who found that full sunlight treatment in *Gingko biloba* leads to a significant increase in flavonol content in leaves and promotes the expression of *PAL*, *CHS*, *F3H*, and *FLS* genes. Concerning transcription levels of genes related to flavone biosynthesis (*CHS* and *CHI*) and flavone production (*FNS*), flavone content is significantly correlated only to *FNS* gene transcription level. This result is symmetrical with that of Yan et al*.* (2014), who studied flavone (apigenin) accumulation and biosynthesis expression of its relative genes, *CHS*, *CHI*, and *FNS*, in celery. They revealed that the expressions of *CHS* and *CHI* are involved in apigenin biosynthesis, but *FNS* expression was specifically associated with apigenin accumulation.

To test the relation between flavonoids production and the phylogeny of the studied plant species, the chloroplast gene *rpl16* intron region was isolated from the studied plant species and sequenced. The chloroplast fragment amplified was 900 bp long and contained 32% GC nucleotides. This ratio of GC coincided with Zhu et al. (2019) and Wang et al. (2020), who found similar percentages of GC content (37.64% and 37.5%) in *Apium graveolens* and *Pimpinella smithii,* respectively. The resultant phylogeny from the chloroplast *rpl16* intron region coincided with the dendrogram and PCA analyses. This coincidence between molecular phylogeny and other analyses such as flavonoids content (with its sub-groups, flavonol and flavone), other factors related to flavonoids production (ABA-sucrose–PAL activity) and gene expression suggests that phylogeny can predict flavonoids content. The agreement of the present molecular data with secondary metabolites (flavonoids) has been approved in several studies, including Choze et al. (2010), Fico et al. (2003), and Wollenweber et al. (2003), which showed that at different hierarchical levels, the occurrence and distribution patterns of flavonoids mostly confirm newer molecular findings. Moreover, Kharazian, (2014) revealed that the chemical diversity of flavonoid profiles in 14 *Salvia* species (flavones, flavonols, flavanones, isoflavones, dihydroflavonols, and chalcones) can be used to assess the taxonomic status of the *Salvia* genus and that flavonoids are suitable indicators.

It was noteworthy that molecular phylogeny in the present study clustered the five species of *P. crispum*, *A. majus*, *C. cyminum*, *Ap. graveolens,* and *D. tortuosa* in one branch apart from the other species. These species have the highest flavone content. Clustering of these plant species with high flavone content with each other may confirm that these plant species (Apium clade of subfamily Apioideae) are more advanced than the other species. Flavones have previously been shown to be almost entirely found in taxa considered to be advanced or more specialized, whereas flavonols predominate in less advanced genera (Zech 1999). The hypothesis that high flavone content species are more advanced is consistent with Salatino (2000), who studied the distribution and evolution of flavonoids in the family Eriocaulaceae and found that primitive groups of Eriocaulaceae were characterized by flavones and flavonols while more advanced groups possess only flavones. This hypothesis is confirmed by the chemical structure, which shows that the hydroxyl group in position 3 in flavonols reduces the inhibitory activity and, therefore, flavones are better polyphenol oxidase inhibitors than flavonols (Halbwirth 2010).

Flavonol synthase is the crucial enzyme in the production of the flavonol. In the present study, the phylogeny of the *FLS* gene of some species of Apiaceae was found to be similar to the phylogeny of the same studied plant species based on chloroplast gene *rpl16*, except for *E. campestere* with low flavonol content and P. crispum with high flavonol content. Analysis of the DNA sequence of the *FLS* gene of the studied high flavonol plant species revealed that the variability in the sequence of high flavonol species is lower than that of low flavonol species. However, the regions of DNA where a cytosine nucleotide is followed by a guanine nucleotide (CpG) were higher in plant species of high flavonol content; they were 1.37-fold higher than low flavonol species. Gene body methylation of transcriptionally active genes is a common feature in various eukaryotes, implying a conserved function and related to gene expression levels (Fang et al. 2010; Zemach et al. 2010).

The current study's insignificant correlation of flavone content with other factors such as ABA hormones, sugars, and transcription level of other genes places the entire potential on the *FNS* gene, which may be a cause in the evolution of this family (Apiaceae). The phylogeny of the *FNS* gene in the studied species was different from their phylogeny based on the chloroplast *rpl16* gene. Moreover, the comparison between the DNA alignments of species containing low and high flavone content showed that the sequence variability of high flavone content species is lower than that of low flavone content species. Also, it showed that CpG is higher in high flavone content plant species than in low flavone content species (1.46-fold), and this may be attributed to an increase in the transcription level of the *FNS* gene. Meanwhile, indels showed low values in plant species with high flavone content relative to those of low flavone content species when the indels number in each species is compared with the lowest flavone content species, *D. carota*. It was also found that indel values in the high flavone content species; *P. crispum* and *C. cyminum,* are equal to those in low flavone content species. This result could be an indication that the evolution of this *FNS* gene is different between studied plant species, revealing different mechanisms of evolution.

There are three hypotheses that may explain the overall difference in the phylogeny of the studied species between the *FNS* gene and *rpl16* gene. The first hypothesis is based on the fact that the *FNS* gene has been acquired by duplication of *F3H*. Since *FNS* I has a high degree of similarity (90%) to Apiaceae flavonone 3β-hydroxylase (*FHT*) it was proposed that *FNS I* had evolved from an ancestral *FHT* by gene duplication (Martens et al. (2003). However, the clade of *D. carota* species with low flavone might be the result of the loss of the functional copy of the *FNS* gene or have occurred as a pseudogene which functions with low efficiency (nonfunctional duplicates are referred to as pseudogenes) where studies by Guo et al. (2009) and Zou et al. (2009) showed that nonfunctional duplicates are not always deleted but plant genomes are always littered with thousands of apparently degenerated genes. Although pseudogenes are presumably nonfunctional, a small subset of them has been found to be obviously expressed in rice and Arabidopsis (Thibaud-Nissen et al. 2009; Zou et al. 2009).

The second hypothesis is that highly expressed genes are likely to be more "crucial" to an organism and could be subjected to high selective pressure. This is displayed by the two species of high flavone content, *Ap. graveolens* and *D. tortuosa,* which were separated from the other species and showed a lower number of indels in the sequence of the *FNS* gene relative to those in *D. carota.* This might be a result of the high constraints on these species, which lead to a decrease in the values of indels. This result agrees with Lu and Rausher (2003), who studied three core anthocyanin structural genes: chalcone synthase (*CHS*), anthocyanidin synthase (*ANS*), and UDP-glucose flavonoid 3-O-glucosyltransferase (*UF3GT*) in the genus *Ipomoea*. They found that the mean number of indel-creating events in *CHS* was found to be four times lower than in *ANS* and *UF3GT*. They proposed that highly expressed genes are likely to be more "critical" to an organism and are subject to a higher level of selective restriction rather than a higher frequency of positively selected substitutions.

The third hypothesis relied on the existence of two high-flavone species (*P. crispum* and *C. cyminum*) among low-flavone species. This can be explained in two ways: A. These plant species acquired an additional copy (recent gene duplication), so they did not differ in sequence, but they differed in the number of gene copies (gene dosage). Consequently, they have an increase in the transcription level of flavone content. The principle of recent gene duplication has been reported by several authors (Graur and Li 2000; Jensen 2001). B. The transcription level of the *FNS* gene in these two species, *P. crispum* and *C. cyminum*, increased at the cost of evolution (gene-code sequence bias). Gout et al. (2010) showed that the selective pressure on the evolution of gene dosage is directly dependent on gene expression level. Based on the fact that gene expression is a costly process, they proposed a COSTEX model, predicted that selective pressure against mutations should be stronger in highly expressed genes. Moreover, Ho and Smith (2016) studied the molecular evolution of anthocyanin pigmentation core genes *CHI*, *F3H*, and *DFR* in the family Solanaceae. They found that *CHI* and *F3H* have significantly higher substitution rates in lineages without floral pigmentation, indicating that this increase is due to relaxed restrictions on anthocyanin genes in the unpigmented lineages and revealing that losses of pigmentation are achieved by changes in gene expression as opposed to structural mutations.

## Conclusion

**Fig. 10:**
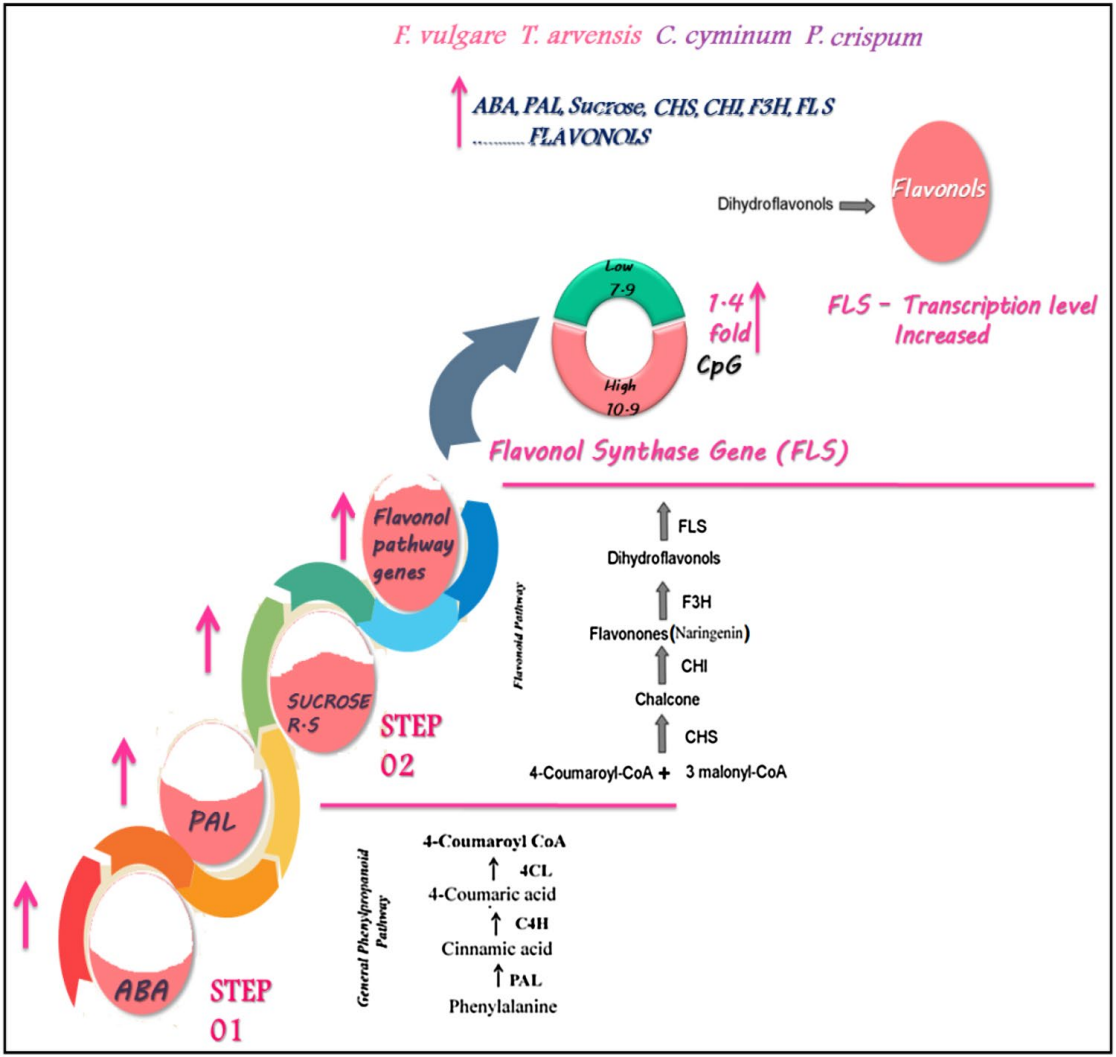
The overall mechanism of flavonol production in the family Apiaceae

**Fig. 11:**
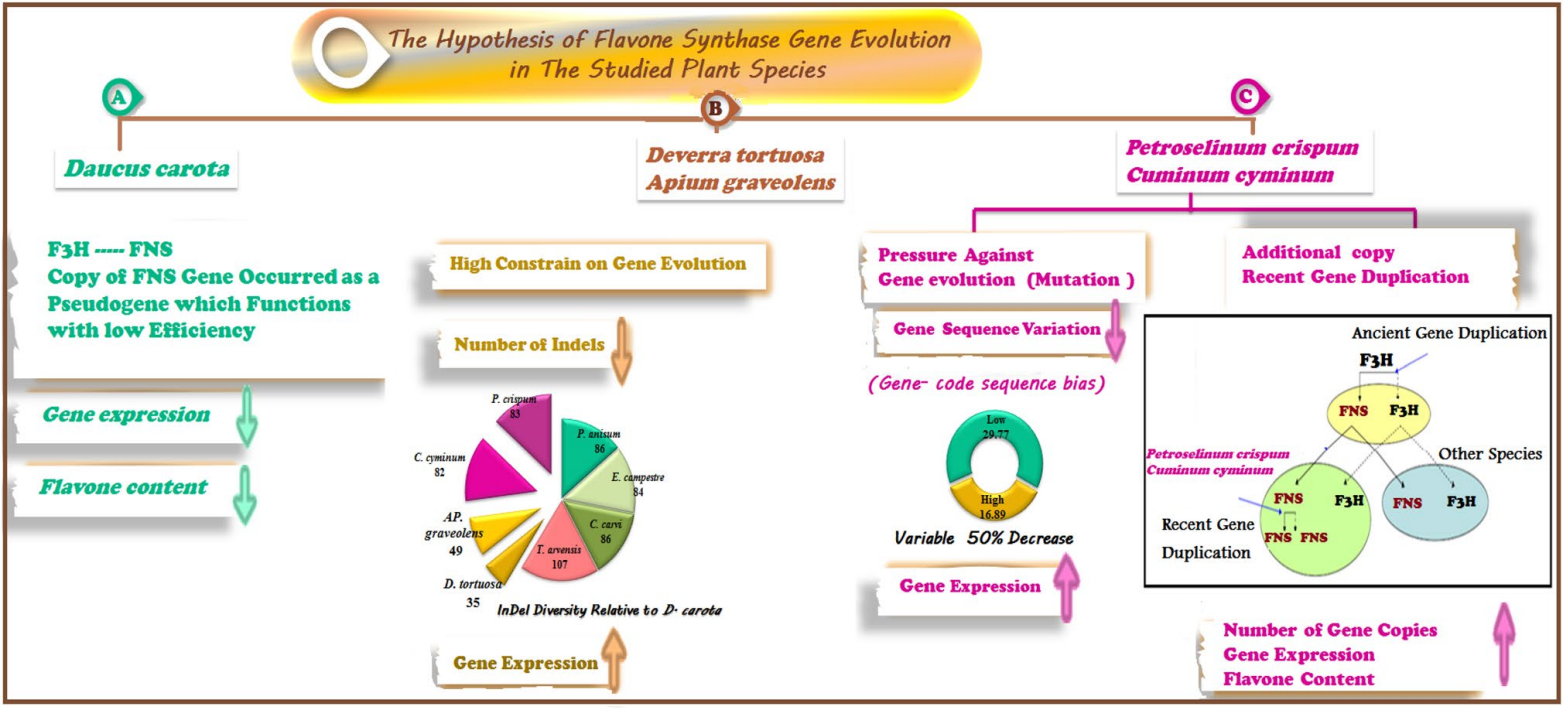
The overall hypothesis of Flavone synthase gene evolution in the family Apiaceae

The current study investigates the hypothesis that the phylogeny of the studied plant species in the family Apiaceae is associated with flavonoid production. The phylogeny of the studied plant species was compatible with the measured flavonoid production as most species of highly flavonoid species clustered with each other; which was identical to the flavone production pattern. This correspondence supports the earlier findings, which revealed that advanced species in the family Apiaceae contained flavone while primitive ones did not. Given that the clade of subfamily Apioideae, which includes *P. crispum*, *C. cyminum, Ap. graveolens*, *A. majus*, and *D. tortuosa*, is more advanced, it could be assumed that any species in this clade contains a high amount of flavone content. However, the phylogeny of the key gene of flavone, *FNS*, was not consistent with its production, whereas *FNS* was highly restricted to natural selection as a cost of higher transcription levels (gene sequence-codon bias). The flavonol content could be increased or regulated by many variables; it could be induced by different concentrations of ABA, sucrose, elevated PAL activity, and high transcription levels of *CHS*, *CHI*, *F3H*, and *FLS* genes (Fig. 10). Meanwhile, flavone content depends mainly on the evolution of the *FNS* gene and its structure, and may thus aid in future studies to increase the flavone content in certain species by transferring the *FNS* gene from those high-flavone species to another species with low flavone content (Fig. 11).

## Supplementary Information

Below is the link to the electronic supplementary material.

Supplementary file1 (PDF 1480 kb)

## Data Availability

Not Applicable.
