# Supplementary material for: Molecular phylogenetic study of flavonoids in medicinal plants: a case study family Apiaceae
Source: J Plant Res. 2023 Feb 28;136(3):305–22. doi: 10.1007/s10265-023-01442-y (PMC10126080; doi:10.1007/s10265-023-01442-y)
Supplement: Supplementary file 1 — Supplementary file1 (PDF 1480 kb) [file 10265_2023_1442_MOESM1_ESM.pdf]

Journal of Plant Research

Molecular Phylogenetic Study of Flavonoids in Medicinal Plants: A Case Study Family Apiaceae

Dalia Youssef [1], Ranya El-Bakatoushi [1], Asmaa Elframawy [2], Laila El-Sadek [3], Ghada El Badan[3]

[1]Biology and Geology Sciences Department, Faculty of Education, Alexandria University, Alexandria, El-shatby, 526 Egypt
[2]Nucleic Acids Research Department, Genetic Engineering & Biotechnology Research Institute (GEBRI), City for Scientific Research and Technological Applications, Alexandria, Borg El-Arab, 21933 Egypt
[3]Botany and Microbiology Department, Faculty of Science, Alexandria University, Alexandria, Camp Caesar, 21525 Egypt

Corresponding author

Dalia Youssef

[1]Biology and Geology Sciences Department, Faculty of Education, University of Alexandria, EgyptAlexandria, 21526 El-shatby, Egypt

E-mail : DaliaYoussef@alexu.edu.eg shahd2000@live.com

Tel. 01066491833

ORCID: **0000-0002-1023-3567**

Sequences and analysis of rpl16 gene in the studied plant species

D. carota **MW036658**

CTGCAGTTGATGGTTGGTTCTGATTCCATCTCTACTACAGAACTGGACATGAGAGTTTCTTCTCATCCAGC
TCCTCGCGAATAATCAAATTTTCAAAATGTCTATTATCATTTGTATATCTTGCTTTTTTAGCTAACTAAAC
ATATTAATTTTTTTTTATTTTAATATAGTATTATTATTTTATTTTTTTCTTTTTATCCTATTGGATTGAGC
AAAAATTATCAATCCAAGAAGATAAAGTTTTCACGGGCGAATATTGACTCTTTTCGTCGCTATTTTAGTTG
TAGGGTTAACTCAGACTTTTCTTTTCCCAATAGATGAAAGAACTAGTCTCTGGTTTGTTTCGCCATCCCGA
TCAATGAGTCTTATTTCAATAGATTTGAATTCACAGGTTCATCGTTCCCATCGCTTCTTACTTAATGGTTA
GGTCTTATTTCTACAATGGAGCTCATAATTAACTTTTTTCTTGAGCCGATTTTCTTAGTCTTTATTGGCTT
GAAGCTCTTATTTTTTTTTGTTCTATGAACGGATTCATTTTGTTTTTTATAATCCATATTGATTGATGCTTT
ATATTACATTGCTTTTTTATGAGACTTTTGAGATGACTCATAAACCTTACATATTGGATGGAATTTTATAT
CGTTGTTTTTCTCCCCTTCTTTCACCCTCCCGTTTATCCACATCTTTCTTCTTCTCTTCACAACTTAGAAT
CATATTTTTTCTTTTGTTTATGCAAAAAAGATTTCAGTTGCTACAGTGATATGACTGATATATCATATCTT
GACTGTTTTTTGGATCCAGATAATGTGAAGTGATGAGTTGGTTATTAGTTCTATAGTTATAGTTATTTGTT
TATACAAAAAAGCTGGTCTTTTTTTTTTCTTTAATCCTACCCCAAAAAACCAACG

P. anisum **MW036659**

TGCTGCTGATGGTTGGTTCTGATTCCATCTCTACTACAGAACTGGACATGAGAGTTTCTTCTCATCCAGCT
CCTCGCGAATAATAAAATTTTCAAAATGTCTATTATTCATTTGTATATCTTGCTTTTTTACTAATTAAGCA
TATTAATATTTCTATTTTAATTTTATGTTTTTAAATTGATATTTTTAAATATCATATTCTTTTTTTATGTT
CTAACGAATATTTGTTTTGTTTTTTATCCTATTGGATTGAGCAAAAATTATCAATCCAAGAAGATAAAGTT
TTCACGGGCGAATTTGACTCTTTTCGTTGCTATTTTAATTTTAGTTGTAGGGTTAACTCATAACTTTTATT
TTCCCAATAGATCAAGGAATTAGTGCTGGTTTGTTTCGCCATCCCGATCAATGAGTCTGTTTTCAATAGAT
TTGGATTCACAGGTTCCATCGTTCCCATCGCTTCTACTTAATGGTTAGGTCTGATTTCTACAACGGAGCTC
ATAATGAAATTTTTTCTTAAGCCAATTTTCTTAGTCTTTATTGGCCGAAGCTCTTATTTTTTTTGTTCTAT
GAACGGATTCGTTTTCTTTTTTATGAATCCATATTAATTGATGCTTTATTACATTGCTTTTTATGAGATGA
CTCATAAACCTTACATATTGGATCGAATTTTATATCGTTGTTTTTGTTTTTTCTCCCCTTCTTTCACCCTC
CGTTTATCCACATCTTTCTTCTTCGCTTCACAATTTGGATCCTATTTTGTTCTTTTGTTTATGCAAAAAAG
ATTTCAGTTCTACAGTGATATGACCAATATAGCATATCTTGACTGGTTTTTTGGATCCAGATAATGTGAAG
TGATGAGTTGGTTATTAGTCTATAGTTATTTGTTTATACAAAAAAAGGCTGGTCTTTTTTTTTTTTTATCCA
AAAACTAATTACCTAAA

E. campestre **MW036660**

TGCTGTTGATGGTTGGTTCTGATTCCATCTCTACTCAGAACTGGACATGAGAGTTTCTTCTCATCCAG
CTCCTCGCGAATAATAATAAATTTGAATATTTGATTATTCATTTCTATACTTGTTTTTAGATAACTAA
ACATATTAATATTTCTAATTTCAATTAAATTTTGAAATATTGTATTCTTTTTTCTTTTTTATAAGTTC
TAACGAATCTTTATTTTGTTTTGCCTTTTATCTATTGGATTGAACAAAAATTATCAATCCAAGAGAAT
AAAGTTTTCGGGCGAATATTTACTCTTTTCGTCGCTATTTCGGTTGTAGGGTTAGCTCATGACTTTTC
CCAATAGATGAATGAATTTTTATGGTTTGTTTCGCCATCCCGATCAATGAATCTGTTTCAATAGATTT
GGATTCACAGGTTCCATCGTTCCCATCGCTTCTACTTAATGGTTAGGTCGTATTTCTACAATGGAGCC
ATAATGAAATTTGTTCTTGAGCCAATTTTCTTAGTCTTTATTGGCTGAAGCTCTTATTTTTTGTTCTA
GGAACAGATTCATTTCTTTTTTACGAATCCGTATTAATGCTTTATTACACTGCTTTTTATTTATATAT
ATATGAGATGACTCATAGACCTTACATTTTATGGAATTTTATCTCATTGGTTTTGTTTTTTCTCCCTT
TCTTCACCCTCCCATTTATCCACATCTTTCTTCTCTATTTGCTTCACAACTTAAAATCGGCGTAGAAT
CAGATTTCTTTTTCTTTTGTTATGCAAAAAATATTGCAGTTGCTACAGTGATATATCGATATATCATA
TCTTGACTGGTTCTTTGGATCCAGG

Fig. S1 Sequences of *rpl16* gene (intron region, forward) in the studied plant species, and *Aralia spinosa* and *Pittosporum dallii* as an outgroup species.

***An. Graveolens* MW036661**

TGCATGTTGATGGTTGGTTCTGATTCCATCTCTACTACAGAACTGGACTGAGAGTTTCTTCTCATCCA
GCTCCTCGCGAATAAGAAAATTTTAAAAATGTCTATTATTCATTTGTATATCTTGCTTTTTTGCTAAC
TAAGCATATTAATTATTAATTATATTTTTAATATTTTTAAATATCATATTCTTTTTTATGTTCTAACG
AATATTCGTTTGTTTTGCTTTTTATCCTATTGGATTGAGCAAAAATTATCAATCCAAGAAGATAAAGT
TTTCACGGGCGAATATTGACTCTTTCGTCACTATTTTAGTTGTAGGGTTAACTCATGACTTTTATTTT
CCCAATAGATGAAGGAATTAGTCTCTGGTTTGTTTCGCATCCCGATCAATGAGTCTGTTTTCAATAGA
TTTGGATTCACAGGTTCCATCGTTCCCATCGCTTCTTACTTAATGGTTAGTCTGATTTATACAACGGA
GCTCATAATGAAATTTTTTCTTGAGCCAATTTTCTTAGTCTTTATTGGCTCGAAGCTCTTATTTTTTG
TTCTATGAACGGATTCGTTTTTTTTTTATGAATCCATATTGCTTGATGCTTTATTACATTGCTTTTTT
ATGAGATGACCATAAACCTTACATATTGGATGGAATTTTATATCGTTGTTTTTTCTCCCCTTCTTTCA
CCCTTCCGTTTATCCACATCTTTCTCTTCGCTTCACAACTTAGAATCAGATTTATTTTTCTTTTGTTT
ATGCAAAAAAGATTTCAGTTGCTACAATGATATGACCAAATATCATATCTTGACTGGTTTTTTGGATC
CAGATAATGTGAAGTGATGAGTTGGTTATTAGTTCTATAGTTATTTGTTTATAAAAAAGGCTGGTCTT
TTTTTTTTTTATCCTATAACCCTAAAAAAT

***C. carvi* MW036662**

CGCAGCTGATGGTTGGTTCTGATTCCATCTCTACTACAGAACTGGACAGAGAGTTTCTTCTCATCCAG
CTCCTCGCGAATAATAAAATTTTCAAAATTTCTATTATTCATTTGTATATCTTGCTTTTTTAGCTAAC
TAAGCATATTATATTTCTATTTTTCAATTGATATTTTTAAATATCATATTCATATTCTTTTTTATGTT
CTAACGAATATTGGTTTTGTTTTTTATCCTATATGGATTGAGCAAAAATACTCAATCCAAGAAGATAA
AGTTTTCACGGGCGAATATTGACTTTTCGTCACTATTTTAGTTGTAGGATTAACTCATGACTTTTATT
TTCCCAATAGATGAAGGAATTAGTCTCTGGTTTGTTTCGCCATCCCGATCAATGAGCTGTTTTCAATA
GATTTGGATTCACAGGTTCCATCGTTCCCATCGCTTCTACTTAATGGTTAGGTCTGATTTCTACAACG
GAGCTCATAAGTTTTTTATAAATCCATATTGATTGATGCTTTATTACATTGCTTTTTTATGAGATGAC
TATAAACCTTACATATTGGATGGAATTTTATATCGTTGTTTTTGTTTTTTCTCCCCTTCTTTCACCCT
TCCGTTTATCCATATCTTTCTTCTTCGCTTCACACTTAGAATCATATTTTATCATTGTTTATGCAAAA
AAGATTCAGTTGCTACAGTGATATGACCAATATATCATATCTTGACTGGTTTTTTGGATCCAGATAAT
GTGAAGTGATGAGTTGGTTATTAGTTCTATAGTTATTTGTTTATACAAAAGGCTGGTCTTTTTTTTTT
ATTAATCCTATAACCCTAAAACAT

***C. sativum* MW036663**

TGCTGCTGATGGTTGGTTCTGATTCCATCTCTACTACAGAACTGGACAGAGAGTTTCTTCTCATCCAGCTC
CTCGCGAATAATAAAATTTTAAAAATGTCTATTTTCATTTGTATATCTTGCTTTTTTAGCTAACTAAGCAT
ATTTTATTTTATATTTTAAATTGATATTTTTAAATATCATATTCTTTTTTTTCTGTTCTAACGAATATTTGT
TTGTTTTTCTTTTTATCCTATTGGATTGAGCAAAAATTATCAATCCAAGAAGTAAAGTGTTCACGGGCGAA
TATTGACTTTTTCGTCGCTATTTTAGTTGTAGGGTAACTCATGACTTTTATTTTCCCAATAGATGAAGGAA
TTAGTCTCTGGTTTGTTCGCCATCCCGATCAATGGTCTGTTTTCAATAGATTTGGATTCACAGGTTCCATC
GTTCCCATCGCTTCTTACTTAATGGTTAGGTCTGATTTCTACAACGGAGCTATAATGAAATTTTTTCTTGA
GCCAATTTTCTTAGTCTTTATTGGCTCGAAGCTTTATTTTTTTGTTCTATGAACGGATTCGTTTTATTTTT
ATGAATCCATATTGATTGATGCTTTATTACATTGCTTTTTTATTTTTTATGAGATGACTCGTAAACCTTAT
ATTGGATGGAATTTTATATCGTTGTTTTTGTTTTTTCTCCCCTTCTTTACCCTCCGTTTATCCACATATTT
CTTCTTCGCTTCACAATTTAGAATCAGATTTATTTTTTTTGTTTATGCAAAAAAGATTTCAGTTGCTACAG
TGATATGACCAATATACATATCTTGACTGGTTTTTTGGATCCAGATAATGTGAAGTGTGAGTTGGTTATAG
TTTTATAGTTATTTGTTTATACAAAAAAAAAGGCTGGTCTTTTTTTTTTATTAATCCTATAACCCTAAAAAAC
CG

Fig. S1 (continued) sequences of *rpl16* gene (intron region, forward) in the studied plant species, and *Aralia spinosa* and *Pittosporum dallii* as an outgroup species.

***F. vulgare* MW036664**

CCTAGCCGAACCTTGCAGGGTTGGTTCTGATTCCATCTCTACTACAGACTGGACATGAGAGTTTCTTC
TCATCCAGCTCCTCGCGAATAAGAAAATTTTCAAAATGTCTATTATTCATTTGTATATCTTGCTTTTT
TAGCTAACTAACATATTAATTATTAATTCTATTTTTATTTAAATATCATATTCTTTTTTATGTTCTAA
CGAATATTCGTTTTGTTTTGCTTTTTATCCTATTGGATTGAGCAAAAATTATCAATCCAAGAAGATAA
AGTTTTCACGGGCGAATATTGACTCTTTCGTCACTATTTTAGTTGTAGGGTTAACTCATGACTTTGAT
TTTCCCAATAGATGAAGGAATTAGTCTCTGGTTTGTTTCGCCATCCCGATCAATGAGTCTGTTTTCAA
TAGATTTGGATTCACAGGTTCCATCGTTCCCATCGCTTCTTACTTAATGGTTAGGTCTGATTTATACA
ACGGAGCTCATAATGAAATTTTTTCTTGAGCCAATTTTCTTAGTCTTTATTGGCTCGAAGCTCTTATT
TTTTTGTTCTATGAACGGATTCGTTTTTTTTTTTTTTATGAATCCATATTGCTTGATGCTTTATTACA
TTGCTTTTTTATGAAATGACTCAAAAACCTTACAATTGGAGGGAATTTTATATCGTTGTTTTTTCTCC
CCTTCTTTCACCCTTCGTTTATCCACATCTTTCTTCTTCGCTTCACAACTTAGAATCAGATTTATTTT
TCTTTTGTTTATGCAAAAAAGATTTCAGTTGCTACAATGATATGACCAATATCATATCTTGACTGGTT
TTTTGGATCCAGATAATGTGAAGTGATGAATTGGTTATTAGTTCTATAGTTATTTGTTTATACAAAAA
GGCTGGTCTTTTTTTTTG

***D. tortuosa* MW036665**

TGCAGTTGATGGTTGGTTCTGATTCCATCTCTACTACAGAACTGGACATGAGAGTTTCTTCTCATCCA
GCTCCTCGCGAATAAGAAAATTTTAAAAATGTCTATTATTCATTTGTATATCTTGCTTTTTTAGCTAA
TTTATATTTTTAATATTCTATTCTTTTGTTCTGTTCTATCTAATATTCGTTTTGTTTTGTTTTTTATT
GTATTGTATTGTATTGGATTGATCAAAACTTAAAAATCCAAGAAGATAAGGTTAACATGGACTCTTAT
CGACTCTTTTCTTCACGATTGGATTTGAGGGACAACTCATGACTTTTATTTTCCCAGAATAAGAAGGA
AGTATGCTCCGCCTTGTTTCGCCATCCCGATGATTGCAACTGATTTGGATACATTTGATTCACAGGCC
CCATCCTTCCCATCGCTTCTTACTTAATGATTAGGTCTGATTTCTTCAACAGAACTCATATTGAAGTT
TTTTCTTGAGCCAATTTTCTTGGTCTTAATTGCTTCGAATCTCGTATTTTTTTGTTCTACGAACGGAT
TCGTTTTTTTTTTTTATGAATCGATACCTTTTGACGTTTTTTTTTTTTGATTTTTTTTAAAAAGACTC
AAAAACCTAAGGAATTGTATGGAATTGTATTTCCTCCCTTTTTTTCCCCTTCCCTTTCCCCCCCATTT
TTCCTCTTCTTTCTTCTTCTTTAACAACATAAAATCAAATTTATTTTTCTTTTGTTAAATCAAAAAAG
ATTCCAGTTGCTGCCCAGATATGACCAACATGACAGATCTTGACGGGCCTATGAAGCCAAATAATGAG
AAGTGAAGAGTTGCTTATGATATCTGTAGAA

***T. arvensis* MW036666**

TAACCCCCGTATAATCTATCTTTCTGGTTGGTTCTGATTCCATCTCTACCCAGAACCGGACATGAGAG
TTTCTTCTCATCCAGCTCCTCGCGAATAATAAAATTTTTAAAAAGTCTATTATCATGCGTATATCTTG
CTTTTTTAGCTACTAAACATATTAATTTTTCTATTTTATATTTTTAAATATCGTATTATTTTTTATGT
TATACCTAATATTTATTTTGGTTTGCTTTTTATCCTATTTGATTGAGCAAAAATTATCAATCCAATAA
GATAAAGTTTTCACGGGCGAATATTACTCTTTTCGTCGCTATTTTAGTTGTAGGGTTAACTCATGACT
TTTCTTTTCCCAATAGATAAAAGAATAAGTCTCTGGTTTGTTTCGCCATCCCGATCATGAGTCTGATT
TCAATAGATTTGAATTCACAGGTTACATCGTTCCCATCGCTTCTTACTTAATGGTTAGGTCTGATTTC
TACAATGGAGCTCATAATTAAAATTTTTCTTGAGCCGATTTTATTAGTCTTTATTGGCTCGAAGCTCT
TTTTTTTTTTCTATGAACGGATTCGTTTTCTTTTTTATGAATCCATATTTATTGATGCTTTATTACAT
TGCTTTTTTTTGAGACTTTATAAGATGACTCATAACCTTACATATTGGATGGAATTTTATATCGTTGT
TTTTTCTCCCCTTCTTTCACCCTTCCGTTATCCACATCTTTCTTCTTCTCTTCACAACTTAGAATCCG
ATTTTTTTCTTTATGCAAAAAAGATTTCAGTTGCTACAGCAAATGACCGATATATCATATCTTGACTG
GTTTTTTGGATCCAGATAATGTGAAGTGATGAGTTGGTTATTAGTTCTATAGTTATTGTTTATAA

Fig. S1 (continued) sequences of *rpl16* gene (intron region, forward) in the studied plant species, and *Aralia spinosa* and *Pittosporum dallii* as an outgroup species.

***Ap. graveolens* MW036667**

TGCAGCTGATGGTTGGTTCTGATTCCATCTCTACTACAGAACTGGACATGAGAGTTTCTTCTCATCCA
GCTCCTCGCGAATAAGAAAATTTTCAAAAGTCTATTTTTCATTTGTATATCTTGCTTTTTTAGCTAAC
TAAGCATCTTATATTTCTATTTTTAAATTTCTATTTTTAAATATCATATTCTTTTTTTATGTTCTAAC
GAATATTCGTTTTGTTTTGCTTTTTATCCTATTGGATTGAGCAAAAATTATCAATCCAAGAAGAAGAT
AAAGTTTTCACGGGCGAATATTGACCTTTTCGTCACTATTTTAGTTGTAGGGTTAACTCATGACTTTT
ATTTTCCCAATAGATGAAGGAATTAGTCTCTGGTTTGTTTCGCCATCCCGATCAATAGTCTGTTTTCA
ATAGATTTGGATTCACAGGTTCCATCGTTCCCATCGCTTCTTACTTAATGGTTAGGTCTGATTTCTAC
AACGGAGCTCATAATGAAATTTTTTCTTGAGCCAATTTTCTTAGTCTTTATTGGCTCGAAGCTCTTAT
TTTTGTTCTATGAATAGATTCGTTTTTTTTTTTTATGAAACCCTATTGGTTGAGGCTTTATTACATTG
GTTTTTTATTTTTTATGAAATGACTCATAAACCTACATATTGGATGGAATTTTATATCGTTGTTTTTT
CTCCCCTTCTTTCACCCTTCCGTTTATCCACATCTTTCTTCTTCCCTTCACAACTTAAAAACAAATTT
ATTTTTCTTTTGTTTATGCAAAAAAAATTTCAGTTGCTACCGTGATATGCCAATATATCATATCGTGA
CTGGGTTTTTGGATCCCGAAAATGGGAAAGGATGAGTTGGGTATTAATTCTATAATTATTTGTTTAA

***C. cyminum* MW036668**

CCATAGTATCTTGCTGGGTTGGTTCTGATTCCATCTCTACTACAGAACTGGACATGAGAGTTTCTTC
TCATCCAGCTCCTCGCGAATAATAAAATTTTCAAAATGTCTATTATCTTTGTATATCTTGCTTTTTTA
GCTAAATAAACATATTTATTTTTCGATTTTTTTTTAATCGTATTATTATTATTATTTTTTTTTTTTTT
TTCTTTCTTTTGTACCCAATTGAATTGGCAAAATTATCAACCCAAAAAAATAAAGTTTTCACGGGCAA
AAATGGACCCTTTTCGCCCTTTTTTAATTGTAGGGTTAACCCAGGACTTTTCTTTTCACAAAAAATAA
AAAAATTATCCCGGGTTTGTTTCCCCCCCCCAATCAAGGAGTCAATTTCAAAAAATTTGGATTCAAGG
TTCCCTCGTTCCCATCGCTCCTAACTTAAGGGTTAGGTCTGATTTCAACAAGGGGCTCAAAAGGAAAT
TTTTTCTTGACCCAATTTTATTATTCTTTATGGGCCCAAACCTCTAATTTTTTTTCAATAAACGGATT
CTTTTTCTTTTTTAGGCACCCAAATGGATGGAGGTTTATTACATTGCTTTTTTAAAAAACTTTATAAA
ATGACCCAAAAACTTACATATGGAGGGAATTTTTAAATCCGTTTGTTTTTTGTTTTTTTCCCCCCCTT
CTTTCACCCCTTCCCTTTATCCCACATTCTTTTCTTTCTTTCTCTTTCACAAACTTAAAAAATCAAAA
TTTTTTTCTTTTTGTTTTAAGGCAAAAAAAAAAAATTTTCAGTTTGCCAACAGGCGAATATGGACCGAA
TATTATCCAAAATCCTGGGAACGGGGGTTTTTTTTGGGAATCCCAAAATAAATGGTGAAAAGTGGG

***A. majus* MW036669**

TGCAGTTGATGGTTGGTTCTGATTCCATCTCTACTACAGAACTGGACATGAGAGTTTCTTCTCATCC
AGCTCCTCGCGAATAAGAAAATTTTCAAAATGTCTATTATTCATTTGTATATCTTGCTTTTTTAGCTA
ACTAAGCATATTAATATTTAGATTTTCTATTTTTAAATTTATATTTTTAAATATCATATTCTTTTTTT
ATGTTCTAACGAATATTCGTTTGTTTTGCTTTTTATCCTATTGGATTGGATTGAGCAAAAATTATCAA
TCCAAGAAGATAAAGTTTTCACGGGCGAATATTGACTCTTTTCGTCACTATTTTAGTTGTAGGGTTAA
TTCATGACTTTTATTTTCCCACTAGATGAAGGAATTAGTCTCTGGTTTGTTTCGCCATCCCGATCAAT
GAGTCTGTTTTCAATAGATTTGGATTCACAGGTTCCATCGTTCCCATCGCTTCTTACTTAATGGTTAG
GTCTGATTTCTACAACGGAGCTCATAATGAAATTTTTTCTTGAGCCAATTTTCTTAGTCTTTATTGGC
TCGAAGCTCTTATTTGTTTGTTCTATGAACGGATTCGTTTTTTTTTTTTTTTTTTTTTAAAAACCCAA
ATGGGTGGGGGCTTTATAACATGGTTTTTTTAAGAAAGGACCCAAAAACCTTACATTTGGGAGGGAAT
TTTATTTCGTGGTTTTTTCCCCCCTTTTTTCCCCCCTCCGTTAATCCACCCTTTTCTTCTTCGTTTCC
CAATTTAAAAAAAAATTTTTTTTTTTTTTGTTAATGCAAAAAAAATTCAGTTGCTACATGGAAATAAC
CAAAATACCAAATCTGGACGGGC

Fig. S1 (continued) sequences of *rpl16* gene (intron region, forward) in the studied plant species, and *Aralia spinosa* and *Pittosporum dallii* as an outgroup species.

***P. crispum* MW036670**

TGCTGCTGATGGTTGGTTCTGATTCCATCTCTACTACAGAACTGGACATGAGAGTTTCTTCTCATCCAGCT
CCTCGCGAATAAGAAAATTTTCAAAATGTCTATTATTCATTTGTATATCTTGCTTTTTTAGCTAACTAAGC
ATATTAATATTTAGATTTTCTATTTTTCAATTTCTATTTTTAAATATCATATTCTTTTTTTATGTTCTAAC
GAATATTTGTTTTGTTTTGCTTTTTATCCTATTGGATTGGATTGAGCAAAAATTATCAATCCAAGAAGATA
AAGTTTTCACGGGCGAATATTGACTCTTTTCGTCACTATTTTAGTTGTAGGGTTAACTCATGACTTTTATT
TTCCCAATAGATGAAGGAATTAGTCTCTGGTTTGTTTCGCCATCCCGATCAATGAGTCTGTTTTCAATAGA
TTTGGATTCACAGGTTCCATCGTTCCCATCGCTTCTTACTTAATGGTTAGGTCTGATTTCTACAACGGACT
CATAATGAAATTTTTTCTTGAGCCAATTTTCTTAGTCTTTATTGGCTCGAAGCTCTTATTTGTTTGTTCTA
TGAACGGATTCGTTTTTTTTTTTTTTTATAAATCCATATTGCTTGAGGCTTTATTACATTGCTTTTTTATGA
AATGACTCATAAACCTTACATATTGGAGGGAATTTTATATCGTTGTTTTTTCCCCCCTTCTTTCACCTTTC
CGTTTATCCACATCTTTCTTCTTCCCTTCACAACTTAAAATCAAATTTCTTTTTCTTTTGTTTATGCAAAA
AAAATTTCAGTTGCTACATGAAATGACCAATATATCCTATCTTGACTGGTTTTTTGGATCCAGATAC

Aralia spinosa ribosomal protein L16 (***rpl16***) gene, intron; chloroplast **(AF094458.1)**

GTTATAGTTGAGGGTTGGTTCTGAATTCCATCTCTACTACAGAACTGGACGTGAGAGTTTCTTCTCATCCA
GCTCCTCGCGAATAAGAAAATCTTAAACTTCTCTATTATTCGTTTGTATATCTTGTTTTTTTAGATAACTA
AACATATTCATATTTCTATTTTAAATATTGTATGACTTTTTATAATGTTATAACGAATCTTTATTTTGTTT
TGTCTTTTATTTTCTTCGATTGACCCAAATTTAGCAATCCAAGAAAATAAAGGTTTCGCAGGCGAATATTT
ACTCTTTTTATCGCTATTTCAGTTGTAGGGTTAGCTCTTGATTTTTCTTTTCCCAATAGATGAATATGAAT
GAATTAGTCTCTGGTTTGTTCCGCCATCCCGATCAATGAATCCGTTTTCAATAAATTTGGATTCATAGGTT
CCATCGTTCCCATCGCTTCTTATTTAATGGTTAGGTCTGATTTCTACAATGGAGCTCATAATGAAATTTTT
TCGTGAGTCAATCTTCTTAGTCTTTATTGGCTCGAAGCTCTTATTTTTTTATTTTATGAACAGATTCATTT
AATTATGTACGAATCAGCATTGATGCTTTATTACACTGTCTTTTTTTTTATGAGATGACTCATAGACCTTAC
ATATTGGATGGAATTCTATATCATTGGTATTTTTTTCTCTCTTTCTTTCACCCTTCCATTTATCCACATCT
TTGTTCTCTATTTCGCTTTATAACTTAGAATTAGATTTATTTTTCTTTTGTTTATGCAAAAAATATTTCAG
TTGCTACAATGATATGACCGATATATCATATCTTGACTGGTTCTTTGGATCCAGATAATGCGAAGTGATGA
GTTGGTTAGTAGTTCTATAGTTATTAGTTTATACTAAGAGGCTGGTCTTTTTTTTTATTGAATCCTAACCCT
AAAAAAACCAACGAGTCACAC

Pittosporum dallii ribosomal protein L16 (***rpl16***) gene, intron; chloroplast **(AF094469.1)**

GTTATAGTTGATGGTTGGTTCTGAATTCCATCTCTACTGCAGAACTGGACGTGATAATTTCTTCTCATCCA
GCTCCTCGCGAATAATAAAATCGTCAACTTTCATTTCAATTAAGATAGAATTTGAATTCAGATATACGTTT
AGTTAATGAGTAATACACTGAATCGTGGATTTTATTTAATCCACATCACAAATCTATTATTCATTTGTATA
TTTTGTTTTTTTAGATAACTAAACATATTAATATTTCTATTTTAAATTAGAATGTTATAACGAATCTTTTC
TTGTTTTGGCTTTTATCATATTGGATTGACCAAAATTTAGCAATTCAAGAAAATAAAGTTTTCGCGGGCGA
ATATTTACTCTTTCCATTGCTATTTCAGTTCTAGGGTTAGCTCATAACTTGACTTTTCCCAATAGATGAAT
GAATTAGTCTCTGGTTCGTTTCGCCATCCCGAGCAATGAATCCGTTTTCAATAGATTTTGATTCACAGGTT
CCATCGTTCCCATCGCTTCTTACTTAATGGCTAGGTCCGATTTCTACAATGGAGCTAATAATGAAATTTGT
TCTTGAGTCAATCTTCTTAGTCTTTATTAGCTCGAAGCTCTTATTTGTTTTTGTTCTATGAACAGATTCAT
TTTATTATGTACGAATCAATGTTGATGCTTTATTACACTGCCCTTTTTTTATGAGATGACTCATAGACCTTA
CATATTGGATGGAATTTTATATCATTGGTATTTTTTTTCTCTCTTTCTCTCACCCTTCCATTTATCCACATC
TTTCTTCTCTATTTCGCTTCACAACTTACAATCGGATTTTTCTTTTGTTTATGCAAAAAAGATTTCAGTTG
CTACAATGATATGACCGATATATCATATCTTGACCGGTTCTTTGGATCCAGATAATGCAAAGCGATGGGTT
GGTTATTAGTTCTATAGTTATTAGTTTATATTAAGAGGCTGGTCTTTTTTTTTTTTTTATCCTAATCCTAAA
AAAACCAACGAGTCACAC

Fig. S1 (continued) sequences of *rpl16* gene (intron region, forward) in the studied plant species, and *Aralia spinosa* and *Pittosporum dallii* as an outgroup species.

Table S1: The nucleotide composition of sequences of *rpl16* gene (intron region) in the studied the studied plant species of Apiaceae.

Plant species	Flavonoids content groups	length (bp)	Σ GC %	Σ AT%	G	C	A	T
D. carota	Group 1 of the low flavonoids	907	30.87	69.13	13.89	16.98	25.36	43.77
P. anisum		940	30.21	69.79	14.15	16.06	25.53	44.26
E. campestre		841	31.27	68.73	14.15	17.12	25.33	43.40
An. graveolens		914	30.74	69.26	14.55	16.19	25.82	43.44
C. carvi		840	31.07	68.93	14.29	16.79	26.55	42.38
C. sativum		925	30.59	69.41	15.03	15.57	25.30	44.11
F. vulgare		902	31.82	68.18	14.97	16.85	25.17	43.02
D. tortuosa	Group 2 of the high flavonoids	847	32.94	67.06	14.52	18.42	25.62	41.44
T. arvensis		881	30.99	69.01	13.62	17.37	25.54	43.47
Ap. graveolens		883	31.60	68.40	14.72	16.87	25.48	42.92
C. cyminum		881	32.35	67.65	13.39	18.96	28.38	39.27
A. majus		838	32.34	67.66	15.16	17.18	25.78	41.89
P. crispum		848	31.60	68.40	13.92	17.69	24.88	43.51
**Total average**		880.54	31.42	68.58	14.34	17.06	25.74	42.86

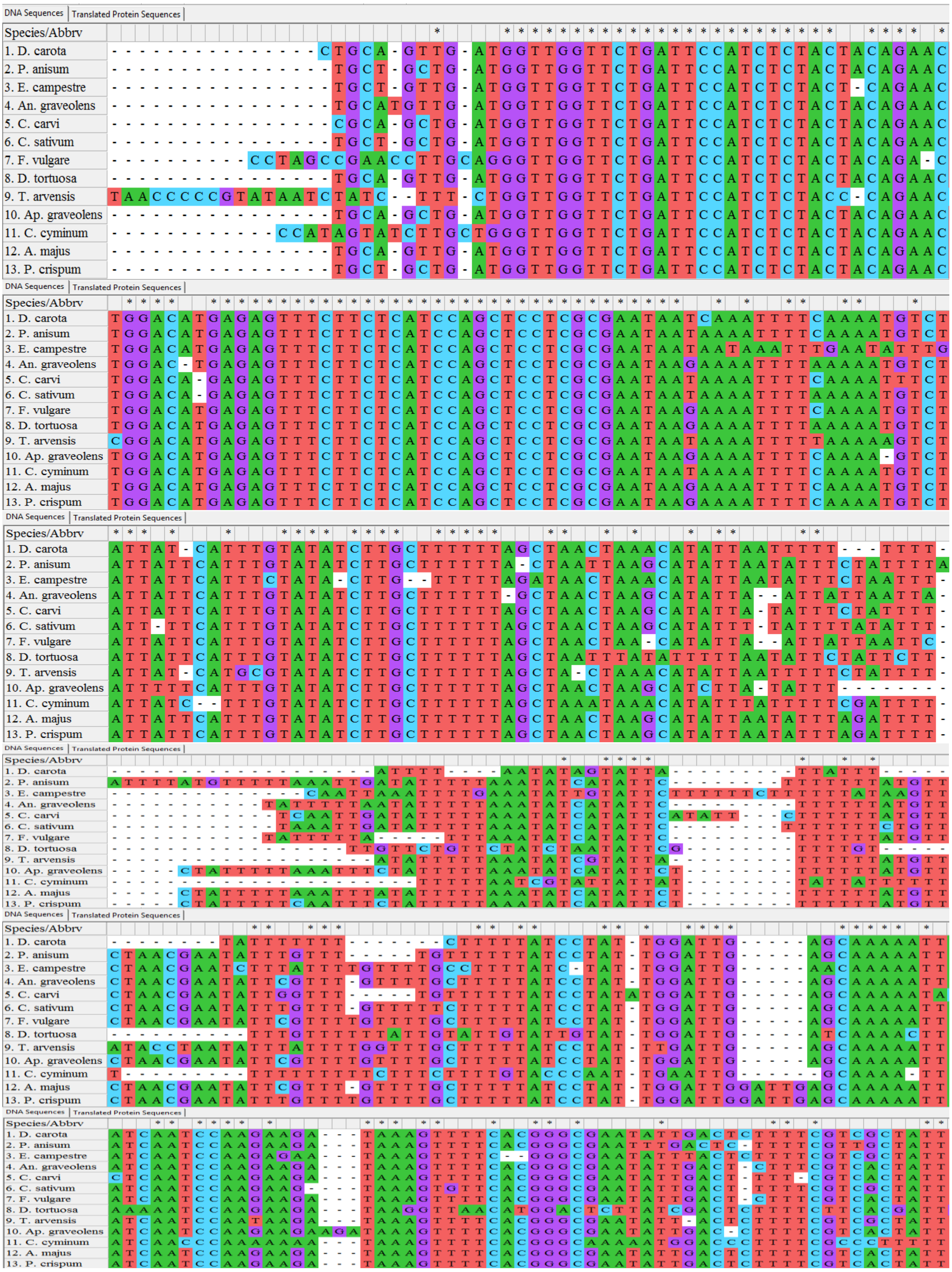

Fig. S2 Alignment of DNA sequences of *rpl16* gene (intron region) isolated from the studied plant species by MEGA-X.

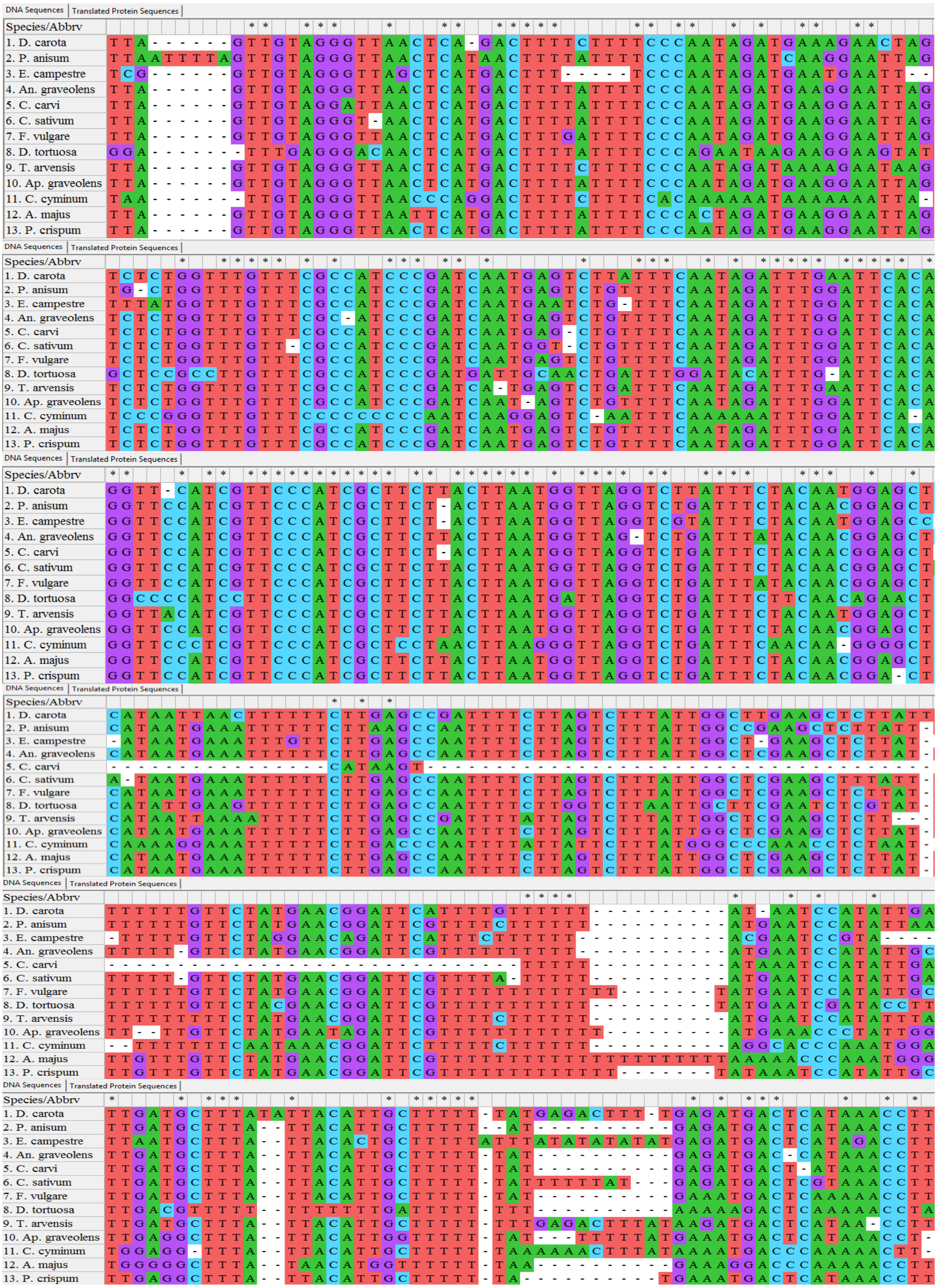

Fig. S2 (continued) Alignment of DNA sequences of *rpl16* gene (intron region) isolated from the studied plant species by MEGA-X.

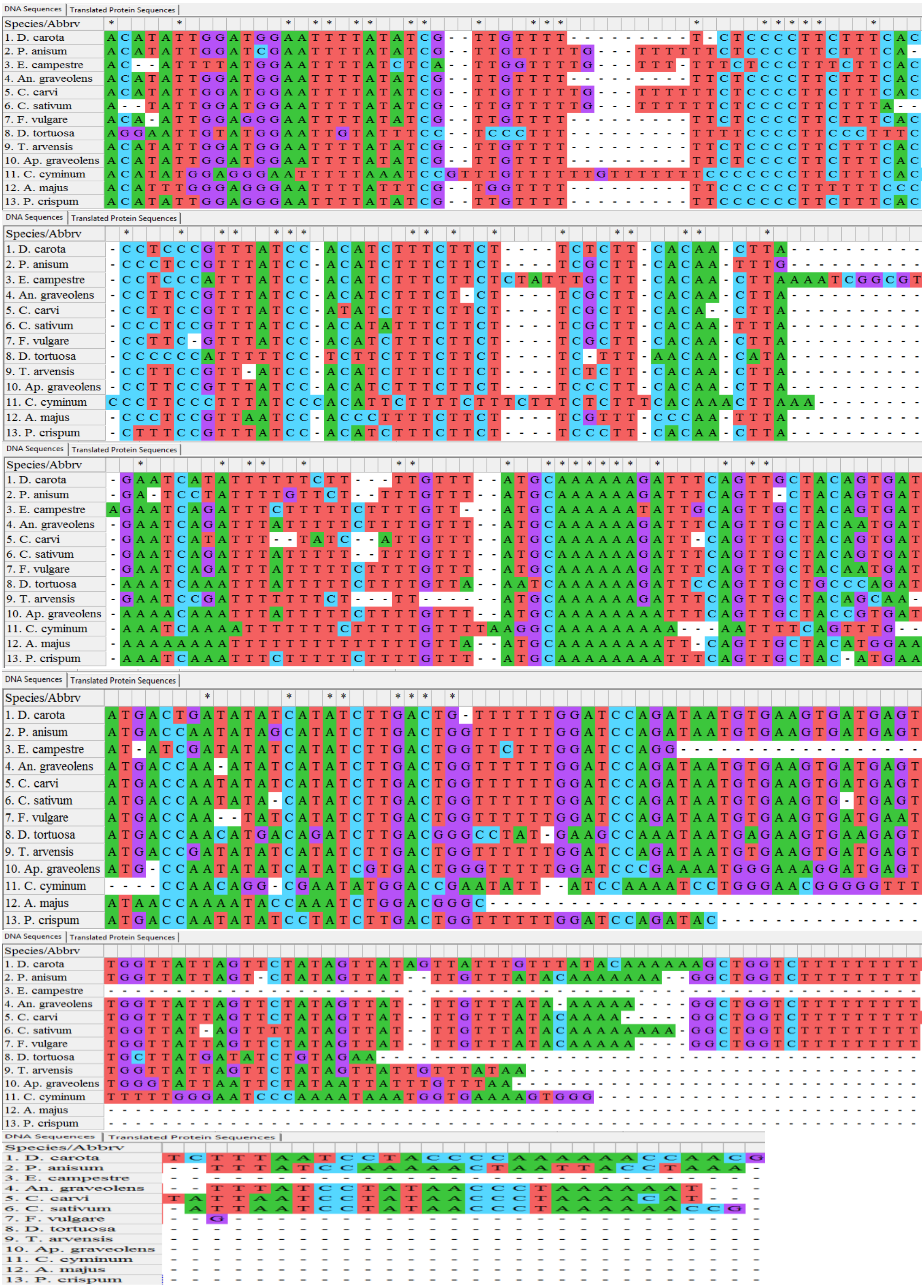

Fig. S2 (continued) Alignment of DNA sequences of *rpl16* gene (intron region) isolated from the studied plant species by MEGA-X.

Sequences and analysis of flavonol synthase gene in the studied plant species

D. carota **MW036671**

AGAAAATTCTACAAGAAGGAGGGGCGGAAAAAGAACAACCTTTCGTTGCTTGTCACAACGAGATGTGGGAA
ACCTGGTTAGATTGATCGCGGAGGCAAGCAAAGAGTGGGGAATATTTCAAGTGGTGAACCATGGCAATATC
GGATGGGCTATTGCAAAGCTACAGAAAGCTGGCAAGGAGTTTTTTGATGCTGCCACAAGAAGAGAAAGACC
TTATTGCTAACCTGAGGGATATCAAGGAGCGGAGGGTTATGGAACATAGCTGCACAAAGAATTGGGACGAC
CACCGGTCTGGTTGATCATTTGCTCCATATCATTTGGCCGAAATCTGCTATTAATTATCAGCTCTGGCCTC
ACAATCCACCCCATTCTCGTCAAACTGACTCATCATTACGCCTCCTTCTCACTTTAGCAGTATTGGTCTTT
CATCACCGCCTCGTAGCTATTTCCATCATATGCTATGCCTAGTCGTTCGAGGAGGAGTTTAAGATTTTCTA
TATTAGTATTTGCTTTGCTCCCTCTCACTCTAACCACTCGTCACTTGCTGTGAGTCGTCCTATGGTTCCTA
TACTGAATTTTCATTATCCTCGGGCGGTGATATCATCTATTTTCACCCTATTTCTATGCTCCCTCCCTTTA
GAAGAAGCTAAAACCATGTAAACCATAGCGTTGGTGTAGATCTATTCTGATA

P. anisum **MW036672**

TTCGTTCTTGGATCATCGAGATGAGGAAACTCTGGTTAGATTGATCGCGGAGGCAAGCAAAGAGTGGGGAA
TATTTCAAGTGGTGAACCATGAATATCGGATGAGGCTATTGCAAAGTTACAGAAAGTTGGCAAGGAGTTTT
TTGAGCTGCCACAAGAAGAGAAAGAACTTATTGCTAAGCCTGAGGGATATCAAGGAGTGGAGGGTTATGGT
ACAAAGTTGCAAAAAGAATTGGGAGGAAAAAAGGGCTGGGTTGATCATTTGTTCCATATCATTTGGCCTAA
ATCTGCTATTAATTATCAATTCTGGCCTAAAAATCCACCTCTTTACAGGTAAACTTACTAATAATTATGCA
GCATTCTTAATTTAGTAGTAAATGTTTTTCTTAAACGGCTTGTTGATATTTACAGTATTATGTTATGCCCT
GTTTTAGAGGATGAGTTTAAAATTTTAAAAATAGTACTTGCTTGCTTACTCAAAAAATATACCAATGAAAA
TTGGCAGAAAAGCAAATGAGGAATATGCCATTGCTTTGAAAGGGGTAGTGGACAAAATATATGAGACCTTG
TTCCTGGGAATTGGGCTTGAGAGGCATGAACTTAAAAGAAACCTCCAGGTGGGTGATGATAAAGTTTACCG
GCTGAAAAATCAATTTATTACCCCTCCATGGCCCTTCGCCCAAATTTAAGCCCTTGGGAATGGTAGGCAC

E. campestre **MW036673**

TTGTTCTTCAGTCCCTGTCATCGATCTCGGATCACCGGATGAGGAAAATCTCGTCAGGTTGATCGCGG
AGGCTAGCGGGAATGGGGAATATTCCAGGTAGTGAACCATGGAATACCTGATGATGCTATTGCCAAGT
TACAGAAAGTTGGAAAGAGTTCTTTGAGTTGCCACAGGAAGAAAAAGAAGTTATTGCTAAGCCTGAGG
GTTACAAAGGTGTGGAGGGTATGGGACAAAGTTACAGAAAGAGGTTGATGGTAAAAAAGGTTGGGTTG
ATCATTTGTTCCATAATATATGGCCTCCCATGCTATCAATTACCATTTCTGGCCTAAAAATCCACCTC
TTTACAGGTCAGTTTGTTTTCTTTCATTTTCTAGTGATTATATAGGCATACACAAACACACACAAAGA
TTGAATATGCTTGCTTTCTTTGTATATATTTTTATTCAGATTCTTCAATTTTACCTCTGCTTGACAAT
CGACAGTCAGACATGGATATGGAAATTGACATTTCGTTTTAAGTTACCATGTCTGATCTTGTATCGAG
TCGGGATAACATAATCTTTCAGTGTATCTGATACATAGTTTTCTGTTTTTTAGACAACGTGTTCTTAA
TTTACTTTAAAATTATCGTATTTGCAGAGAAACAAATGAGGAATATGCCCAAAAACTGAGAGGAGTAG
GAGACAATTGTTTGAGGCCCTATCTTTAGGGGTTGGGCTTGAGAGGCATGAAATGAAAGAAGCTGCAG
GTGGTGATGATATACTTACCTGATGAAGATAAATTACTACCCACCGTGTCCACGTCCAGATCTGGCCC
TCGGAGTTGTGGCCCATACAGTATGTCTGCTATCA

Fig. S3 Partial sequence of flavonol synthase (*FLS*) gene in the studied plant species of Apiaceae and *Lonicera japonica* and *Lactuca sativa* as an outgroup species

C. carvi **MW036674**

CATGGGGTTGTTCTTGAGGTTCCGGTTATCGATCTTGGATCATCGGATGAGGAAACTCTGGTTAGATT
GATCGCGGGCAAGCAAAGAGTGGGGAATATTTCAAGTGGTGAACCATGGAATATCGGATGAGGCTATT
GCAAAGTTACAGGTTGGCAAGGAGTTTTTTGAGCTGCCACAAGAAGAGAAAGAACTTATTGCTAAGCC
TGAGGGATATCAAGGAGGAGGGTTATGGTACAAAGTTGCAAAAAGAATTGGGAGGAAAAAAGGGCTGG
GTTGATCATTTGTTCCATATCATTGCCTAAATCTGCTATTAATTATCAATTCTGGCCTAAAAATCCAC
CTCTTTACAGGTAAACTTACTAATAATTATGAGCATTCTTAATTTAGTAGTAAATGTTTTTCTTAAAC
GGCTTGTTGATATTTACAGTATTATGTTATGCCCTGTTTTAAGGATGAGTTTAAAATTTTAAAAATAG
TACTTGCTTGCTTACTCAAAAAATATACAAATGAAAATTGGCAGAGAAGAAATGAGGAATATGCCATT
GCGTTGAGAGGGGTAGTGGACAAATTATATGAGACCTTGTCTCTGGGGATTGGGCTGAGAGGCATGAA
CTGAAAGAAGCATCAGGTGGTGATGATATAGTTTACATGTTGAAAATCAATTATTACCCACCATCCCT
CGTCCAGATTTAGCCCTTGGAGTTGTGGCACACACTGATATGTCTGCTATCACCATTCTTGTACCTAA
CGAAGAAA

P. crispum **MW036675**

GTTCTTCAGTTCCGGTGATCGATCTCGGATCATCCAACAACGAGGAAAATCTCGTTGAATTAATCGCC
GAGGCAAGCAGAGAGTGGGGTATATTCCAAGTGGTGAACCATGGAATACCAGATGATGCTATTGCAAA
GTTGCAAAAGGTTGGCAAGGAGTTTTTTGAGCTGCCTCAACAAGAGAAAGAAGTCATTGCTAAGCCTG
AGGGGTATCAAGGAGTGGAGGGTTATGGTACCAAGTTACAAAAAGAACTAGGAGGAAAAAAGGGCTGG
GTTGATCATTTGTTCCATATTATTTGGCCTAAATCTGCTGTTAATTTCAATTTCTGGCCAAACAATCC
ACCTCTTTACAGGTAATCTTACTTTTTAGGAGTATTTTGTTCTGCTGCTTCTACGATTATAGAATTAT
ATAATGACATTTCTAATTTAGTAGGAAATATTTTTCTTAAATGGTCTTGTTGAAATGTACAGTATTAG
GCCCTGTTTTACAAGTAGAGTTTAGGAATTCAATAATATACAAAACCTAACAGAAATTTATACTGTCA
AATTCACCTAATATTAACGTGCTCTAGAATTACTGAAGATGATTGTACTTGTAGTGATAATCAAATGT
CTACTTTCTATCTTCTACCTTCAGCTCATTTGAAATTTTAGAAACCACTACTTTATAAGTAACCACAC
AAGATTTATGGAATTGGATGTTACTGTGATGCACTCTTTATATAAAAGTAAAGCACTTGCTACAGCTT
ATGGAACATTTTTAAGTAATATTATCTACAA

T. arvensis **MW036676**

TTCGATCAAAGGACGAGGAAAACCTTGTCAGATTAATCGCGGAGGCGAGCCAGGAGTGGGGAATATTC
CAAGTGTGAATCATGGAATACCGGATGATGCTATTGCCAAGTTACAAAAAGTTGGCAAGGAGTTTTTT
GAGCTGCCACAAAGAGAAAGAACTAATTGCCAAGCCTGAGGGATATCAAGGAGTGGAGGGTTATGGTA
CAAAGTTACAAAAAGAAGCGGAGGAAAAAAGGGGTGGGTTGATCATTTGTTCCATATTATTTGGCCCA
AATCTGCTATTAATTACAATTTCTGGCTAAAAATCCACCTCTTTACAGGTAAGTTTACATTCATTAGT
TCTAGTGCTAGCTTCTACAATTTTAGAATTCTATAACTTCCTAGTTTGGTAGTAAATGTATTTCTAAT
ACGGTCTTGAAATTTACCAGTATATGCTCTGTTTTAGAAGATGAGTTAGAAATTCAAGGATATACAAA
TATGCAAGTACAGTTGTACTGCTATTTTGTTACACGATTACATGAGTACTTGGAAAATCGTACTCTGT
ATAGTTTATCCTCAATGCAATATGTATATATAGTTTTATAGATGTTTCATGTTCTAAACTGGCAAGAA
GCAAACGAGGAATATGCCATAGCGTTGAGGGGGGTAGGGGACAAATTGTATGAAGCCTTGTCTCTGGG
GACGGGCTTGAAGGCATGAACTGAAGAAGCATCAGGTGGTGATGATCTGATTTACTTGTTAAAAATCA
ATTATTACCCACCCTGGCCCTCTCCCGAATATAGCCCCTTG

Fig. S3 (continued) Partial sequence flavonol synthase (*FLS*) gene in the studied plant species of Apiaceae and *Lonicera japonica* and *Lactuca sativa* as an outgroup species

Lonicera japonica flavonol synthase (***FLS***) mRNA, complete cds (**JQ627647.1)**

CCTTGAAAATATCTGCAAGTGAGAGTACTAAAACAATAATGGAGGTTGCAAGAGTACAGCAATAGCGTCAATAAC
AAAATGCATGGACACAATACCATCAGAATATATTAGGTCAGAGAACGAGCAGCCAGCGTCCACAACGTTGCATGG
TGTGCTACTTCAAGTTCCAGTAATTGACATAGACGATAAAAATGTAGTGAAACTCATATCGGATGCTAGCAAAGA
ATGGGGGATATTTCAAGTGATAAATCATGGAATTCCAGATGAGGTAATTGCGAATTTGCAAAAAGTAGGGAAGGA
ATTCTTTGAGGTTGTACCACAAGAGGAGAAAGAGGTGATTGCAAAAACTCCAGGGTCTCAGAATATTGAAGGGTA
TGGTACTTCTTTGCAGAAAGAACTTGAAGGGAAAAGGGGTTGGGTTGATTATTTGTTCCATAAGATTTGGCCACC
TTCTTCTATTGACTACCACTTTTGGCCTAAAAATCCTCCTTCTTACAGAGAAGCCAATGAGGAGTACGCAAATAG
GTTGCAGAAGGTGGCAGACAAGTTGTTAGAGTGCCTTTCATTAGGGCTTGGACTTGAAGGGGGCGAAATTAAAGC
TGCTATTGGTGGGGATGACCTAATTTACCTTATGAAAATCAATTATTACCCACCATGTCCTAGGCCTGACCTGGC
ACTAGGTGTGGTGGCCCACACTGACATGTCAGCAATCACAATTCTTGTTCCCAACGAGGTTCAAGGCCTCCAAGT
CTTTAACAATGACCATTGGTATGATGTCAAGTACATCCCTAATGCCCTTGTCGTTCACATTGGTGATCAAATTGA
GATACTAAGCAATGGAAAATATAAGAGCGTGCTGCATAGAACAACAGTGAACAAAGAGCTGACGAGAATGTCGTG
GCCAGTTTTCCTAGAGCCACCATCGGAGCTCGAAAACGGTCCGATTCCAAAGCTTATAAATGAAGAGAATCCACC
AAAATACAAGACCAAGAAGTACAAAGATTATGTCTATTGTAAGCTTAACAAGCTTCCTCAGTGAAGAATTCTTTA
TTTATATATTAATTAACTAATGCCTTCTATCTTCCTGGTACTACTAAATAATTTATATTTTGTACTATCCTTAC
CAAGAGATATTGTCTTATTATTAATGTCAAGCTTTGGAAGGAATTTGTAATGCTCAATTGACTAATCTGCATTTA
TAATAGAAGAAGTTGGCTCATCTATTACTAAAAAATAAAAAAAAAAA

Lactuca sativa var. crispa ***LsFLS*** mRNA for flavonol synthase, partial cds (**AB359897.1)**

CAATCTTTGAAGCCAGCAAAGATTGGGGGATCTTTCAGGTCGTGAACCATGACATAACAAGTGAAGTCAT
AAGCAAGTTACAAAAAGTTGGTAAAGAGTTCTTCGAGTTGCCACAAGAAGAGAAAGAAGCCATTGCTAAA
CCAGTTGGGTATAAAGGTGTTGAAGGATATGGCACAAAGCTTCAAAAGGAAGTGGAAGGGAAGAAAGGCT
GGGTGGATCATTTGTTTCATAGGGTTTGGCCACCTTCTGCTGTCAACTATCAGTTTTGGCCCAAAAACCC
TCCATCTTACAGGGAAACAAACGAGCAATACACAAGTATGTTGATAGGGGTAGCAAACAAATTGCTTGGA
TTCCTATCAAACGGACTTGGGTTAGAAGAGGGTGAAATAAAACAAGGGTTGGGTGGTGAAGACTTGACCT
ACATGTTGAAAATAAACTACTACCCACCATGCCCATGTCCCGAGCTTGCTCTTGGGGTTGTACCCCATAC
AGATATGTCCTCCATCACCATACTTGTCCCAAATGAAGTTCAAGGTCTACAAGTCTTTAAAGATGGCCAT
TGGTATGATGTTGCATACATCCCTAATGCTCTCATTATTCACATTGGTGACCAAATGGAGATATTGAGCA
ATGGAATATATAAGAGTGTGTATCACAGGGCAACAGTGAACAAAGAGAAGACGAGAATGTCGTGGCCATT
TTTCTTGGA

Fig. S3 (continued) Partial sequence flavonol synthase (*FLS*) gene in the studied plant species of Apiaceae and *Lonicera japonica* and *Lactuca sativa* as an outgroup species.

Table S2: The nucleotide composition of partial sequence of flavonol synthase (*FLS*) gene in the studied plant species of Apiaceae.

Plant species	Flavonol content	length (bp)	Σ GC %	Σ AT%	G	C	A	T
D. carota	Low flavonol content	691	43.85	56.15	21.56	22.29	26.77	29.38
P. anisum		709	38.22	61.78	23.27	14.95	32.44	29.34
E. campestere		851	39.72	60.28	22.56	17.16	28.91	31.37
C. carvi		756	38.62	61.38	23.81	14.81	31.22	30.16
P. crispum	High flavonol content	779	35.04	64.96	19.13	15.92	32.22	32.73
T. arvensis		789	38.78	61.22	22.81	15.97	31.31	29.91
**Total average**		762.5	39.04	60.96	22.19	16.79	30.49	30.54

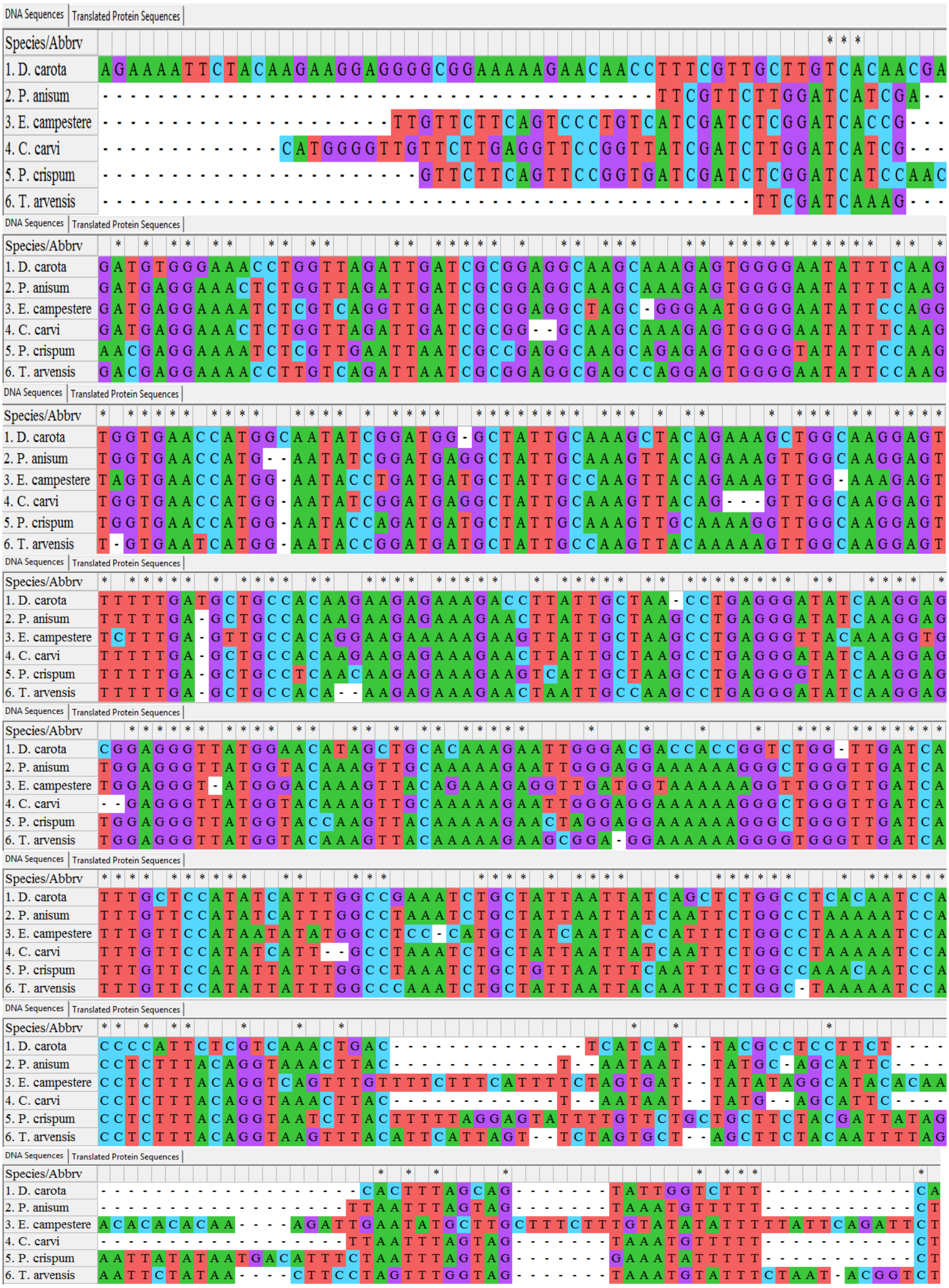

Fig. S4 Alignment of partial DNA sequences of flavonol synthase (*FLS*) gene isolated from the studied plant species by MEGA-X.

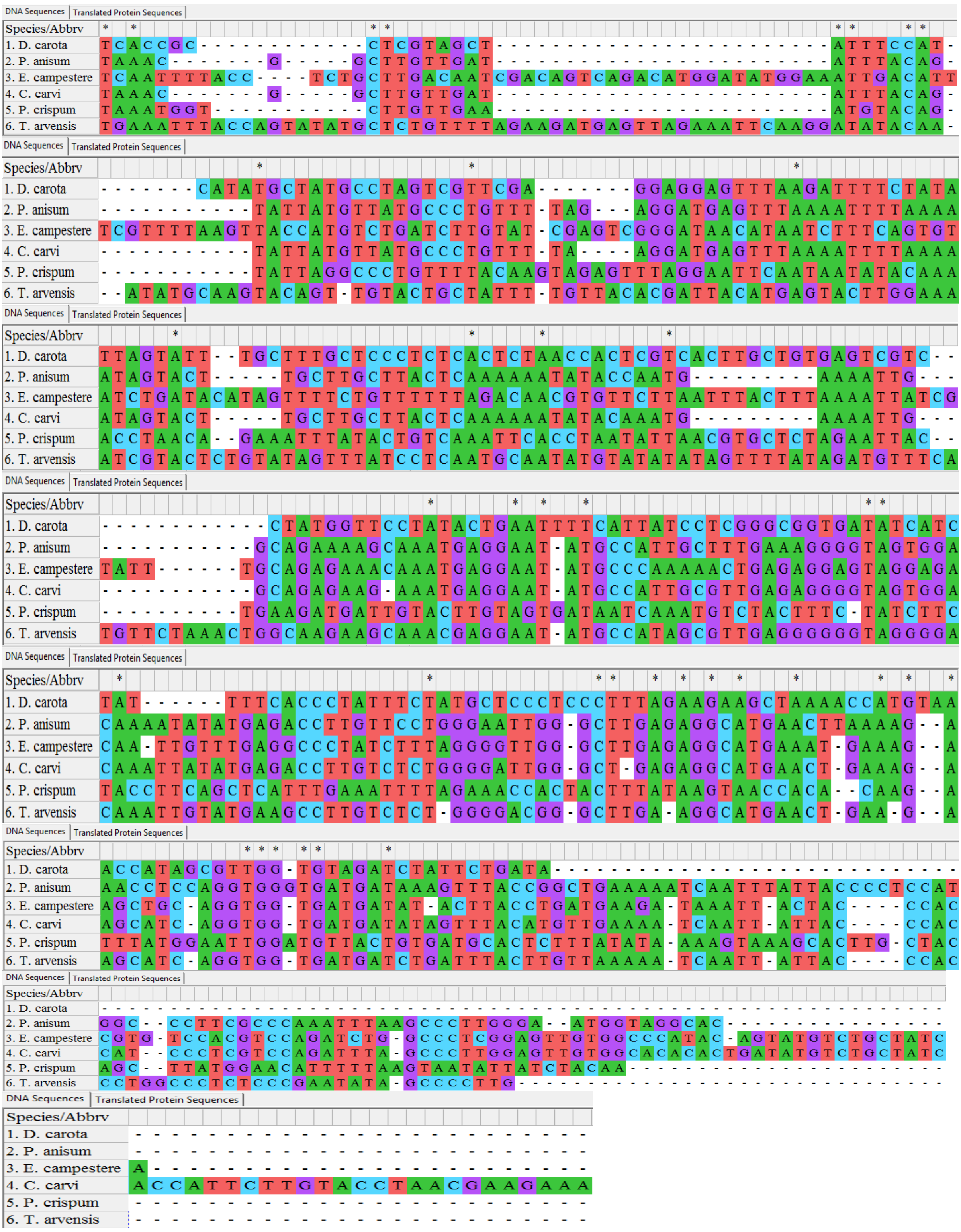

Fig. S4 (continued) Alignment of partial DNA sequences of flavonol synthase (*FLS*) gene isolated from the studied plant species by MEGA-X.

-

Sequences and analysis of flavone synthase gene in the studied plant species

D. carota **MW036677**

CCAAGCTGGTCAATTGGTTCTTTTCTTTTTTTGCTTCAGGATCGCGTTGGTGGATCACCTGCCACAAC
GGCGATGGAGGCCTAACTGGCAATTACTGTTCAGCCTGTGTACGTTGCTATTCTGTGCGTCCTTTTCC
GGAGATGAAGGTAGGCGACGAATGATCTCATACGCTCCTTCAACGGACTCCGTCCACACTACAATTGC
TTATCTCTTGCGAAAGACCAGAGCTCGCAACACTACCTCTTGTGGAGTACGTTGTCTGATTATCCTAG
CCAGGTACATGCGCAGCGGTACAATCAAGCAGCCGATACCTACTGTATCCGTACCGTATACGCTATTC
CTACCTCTACAACTCAGATTGAACCATCGAAATTTCTACATGCCCGTGCATAGCTCCATCCTGTATCC
ATTGAAGATCAGGGAGGGAGGCATCTCCACTCTGGACGTTGTCACCACAGCTCCTCTATAGTATGCTT
ATTAATGTACCACTAGCATCGTATCACTAGTACTTTACTCTGCAAGTGCTGGCTCATTTCACAAAACA
ATGATTGGTGCCCCAGGCATGTACT

P. anisum **MW036678**

CAGAGGCATACGGATCCAGGTACTATTACCATTTTGCTTCAGGACATGGTTGGTGGGTTACAGGCCAC
GAGGGATGGCGGCAAAACCTGGATCACTGTTCAGCCTGTGGAGGGAGCTTTTGTCGTCAATTTGGGTG
ATCATGGTCATGTAAGTTCACTCATTGTTTAAAATGACTATAAGCATATATATTAATTTTGGAATTGA
CCTAACAATTTTGTGGTTATGTGTAGTATTTGAGCAATGGGAGGTTCAAGAACGCGGACCACCAGGCA
GTAGTGAATTCAACCTCAAGCAGATTGTCAATCGCAACTTTCCAGAACCCGGCTCAGAACGCTATAGT
GTATCCATTAAAGATCAGGGAGGGTGAGAAGCCAATTCTGGAGGAGGCCATCACGTACGCGGAGATGT
ATAAGAAAAACATGACTAAACATATTGAGGTGGCTACACAGAAGAAATTGGCCAAGGAGAAAAGATTG
CAAGAAGAGAAGGCCAAGCTGGAAA

E. campestre **MW036679**

CATACGCCATGCGCTCTGGTACTAGCCACATTTTGCTTCACGACCTGGTTGGTGGGTTCAGGCCACGA
GGGATGGCGGCAAAACCTGGATCACTGTTCAGCCTGTGGAGGGAGCTTTTGTCGTCAATTTGGGTGAT
CATGGTCATGTAAGTTCACTCATTGTTTAAAATGACTATAAGCATATATATTAATTTTGGAATTGACC
TAACAATTTTGTGGTTATGTGTAGTATTTGAGCAATGGGAGGTTCAAGAACGCGGACCACCAGGCAGT
AGTGAATTCAACCTCAAGCAGATTGTCAATCGCAACTTTCCAGAACCCGGCTCAGAACGCTATAGTGT
ATCCATTAAAGATCAGGGAGGGTGAGAAGCCAATTCTGGAGGAGGCCATCACGTACGCGGAGATGTAT
AAGAAAAACATGACTAAACATATTGAGGTGGCTACACAGAAGAAATTGGCCAAGGAGAAAAGATTGCA
AGAAGAGAAGGCCAAGCTGGA

C. carvi **MW036680**

TACGGATCCAGGTACTATTACCATTTTGCTTCAGGACATGGTTGGTGGGTTACAGGCCACGAGGGATG
GCGGCAAAACCTGGATCACTGTTCAGCCTGTGGAGGGAGCTTTTGTCGTCAATTTGGGTGATCATGGT
CATGTAAGTTCACTCATTGTTTAAAATGACTATAAGCATATATATTAATTTTGGAATTGACCTAACAA
TTTTGTGGTTATGTGTAGTATTTGAGCAATGGGAGGTTCAAGAACGCGGACCACCAGGCAGTAGTGAA
TTCAACCTCAAGCAGATTGTCAATCGCAACTTTCCAGAACCCGGCTCAGAACGCTATAGTGTATCCAT
TAAAGATCAGGGAGGGTGAGAAGCCAATTCTGGAGGAGGCCATCACGTACGCGGAGATGTATAAGAAA
AACATGACTAAACATATTGAGGTGGCTACACAGAAGAAATTGGCCAAGGAGAAAAGATTGCAAGAAGA
GAAGGCCAAGCTGGA

Fig. S5 Partial sequence of flavone synthase (*FNS*) gene in the studied plant species of Apiaceae and *Lonicera japonica* and *Dahlia pinnata* as an outgroup species.

***T. arvensis* MW036681**

GCATACGGATCCAGGTACTATTACCATTTTGCTTCAGGACATGGTTGGTGGGTTACAGGCCACTAGGG
ATGGCGGCAAAACTTGGATCACTGTTCAGCCTGTGGAGGGAGCTTTTGTCGTCAATTTGGGTGATCAT
GGTCATGTAAGTTCACTCTCTGTTTAAAATGACTATAAACATATATATTAATTTTGGAATTGACCTAA
CTTTTTTGGGGTTATGTGTAATATTTGAACAGTGGGAGGTTCAAGAACGCGGACCACCTCGCAGTAGT
GAATTCAACCTCTTTCATATTGTCGATCGCGACTTTCCAGAACCCGGCTCAGAACGCTATAGTGTATC
CATTAAATATCAGGGAGGGTGATAATCCAATTCTGGAGGAGGCCGTCACGTACGCGAATATGTATAAC
AAAAACATGACTAAACATATTGAGGTGGCTACACAGAAGAAATTGGCCAAGGAGAAAAGATTGCAATA
ATATAAGGCCTCCCTGGAAGATCAGAGAGGGTGAGAAGCCAATCCTGGAGGAGGCCATCACATACGCT
GAGATGTATAAGAAAAACATGACTAAACATATCGAGGTGGCTACCCAGAAGAAATTGGCCAAGGAGAA
AAAGTTGCAAGAAGAGAAGGCCAAGCTGGA

***D. tortuosa* MW036682**

CAGAGGCATACGGATCCAGGTACTATTACCATTCTTCTTCAGGACATGGTTGGTGGTTTACAGGCTAC
TAGGGATGGTGGCAAAACTTGGATTACTGTTCAGCCTGTGGAGGGAGCTTTTGTTGTCAACTTGGGTG
ATCATGGTCATGTACGTACGTTCACCCTCCTTTTTTCATCACTAATATTAAACACATTACACACACAC
ATGCATGCATATATACCATTTTTGCTTCTGAGGAGACATGTAGCCTGGTAATAAATGAGCTAGGTCTA
GGTGCGTGTGTAAAACCTCTAAAGTGAGAGCTCTTGCTAGCTGCTGCATTTATACTTCATCGTTTTAA
GAATGATTATTTTATATGAGCATATATTTTAATTGGCAATTGAGCTGGCAAGTTTGTGCTTGTGTGTA
GTATTTGAGCAATGGAAGATTCAAGAACGCTGACCACCAAGCAGTAGTGAATTCAAGCTCTAGCAGAT
TGTCAATTGCAACTTTCCAGAACCCGGCACAGAATGCGATAGTATATCCATTGAAGATCAGGGCGGGA
GAGAAGGCAGTTCTGGATGAGGCCATCACCTACGCTGAAATGTATAAGAAAAACATGACTAAACATAT
TGAGGTGGCTAACCTGAAGAAATTGGCCAAGGAGAAAAAGTTGCAAGAAGAGAAGGCCAAGCTGGAGA
T

***Ap. Graveolens* MW036683**

TACGTGATCCAGGTACTATTACCATTCTGCTTCACGACATGGTTGGTGGTTTACAGGCTACTAGGGAT
GGCGGCAAAACTTGGATCACTGTTCAGCCTGTGGAGGGAGCTTTTGTCGTCAATTTGGGTGATCATGG
TCATGTAAGTTCGCTCTCTCCCTCTCTGACTATATTTTTGCTTTTAATTTTGGAATTGACCTAACTGT
TTGTGGATATGTGACTCCTATCTGCTGGGGAGGTTCTTAAACGCGGACCAATGATTGATTTTGAATGA
ACCTCAAGCAGATTTCAGAATTTACTTTCCAAATCCCGGCTCTTAACGCTATAATGTATCCATTGGAA
ATCAGGGAGGGGGAGAAGCCAATTCTGGAGGAGGCCATCACTTCCGCGGATATGTATAAGAAAAACTT
GACTAACCCTATTGAGGAGGCTACACAAAATAAATTGGCCATCGAGAAAGGATTGCAGGAAAATAAGG
CATGACTGGATCACCTACGCTGAAATGTATAAGAAAAACATGACTAAACATATTGCGGTGGCTACCCA
GAAGAAATTGGCCAAGGAGAAAAGGTTGCAAGAAGAGAAGGCCAAGCTGG

***C. cyminum* MW036684**

GAGGCATACGGATCCAGGTACTATTACCATTTTGCTTCAGGACATGGTTGGTGGGTTACAGGCCACGA
GGGATGGCGGCAAAACCTGGATCACTGTTCAGCCTGTGGAGGGAGCTTTTGTCGTCAATTTGGGTGAT
CATGGTCATGTAAGTTCACTCATTGTTTAAAATGACTATAAGCATATATATTAATTTTGGAATTGACC
TAACAATTTTGTGGTTATGTGTAGTATTTGAGCAATGGGAGGTTCAAGAACGCGGACCACCAGGCAGT
AGTGAATTCAACCTCAAGCAGATTGTCAATCGCAACTTTCCAGAACCCGGCTCAGAACGCTATAGTGT
ATCCATTAAAGATCAGGGAGGGTGAGAAGCCAATTCTGGAGGAGGCCATCACGTACGCGGAGATGTAT
AAGAAAAACATGACTAAACATATTGAGGTGGCTACACAGAAGAAATTGGCCAAGGAGAAAAGATTGCA
AGAAGAGAAGCCCAAGCTGGAAATA

Fig. S5 (continued) Partial sequence of flavone synthase (*FNS*) gene in the studied plant species of Apiaceae and *Lonicera japonica* and *Dahlia pinnata* as an outgroup species.

***P. crispum* MW036685**

TGCGCTCCTGGTACTATTACCATTTTGCTTCCCGACATGGTTGGCGGGTTACAGGCCACTAGGGATGG
CGGCAAAACCTGGATCACTGTTCAGCCTGTGGAGGGAGCTTTTGTCGTCAATTTGGGTGATCATGGTC
ATGTAAGTTCACTCATTGTTTAAAATGACTATAAGCATATATATTAATTTTGGAATTGACCTAACAAT
TTTGTGGTTATGTGTAGTATTTGAGCAATGGGAGGTTCAAGAACGCGGACCACCAGGCAGTAGTGAAT
TCAACCTCAAGCAGATTGTCAATCGCAACTTTCCAGAACCCGGCTCAGAACGCTATAGTGTATCCATT
AAAGATCAGGGAGGGTGAGAAGCCAATTCTGGAGGAGGCCATCACGTACGCGGAGATGTATAAGAAAA
ACATGACTAAACATATTGAGGTGGCTACACAGAAGAAATTGGCCAAGGAGAAAAGATTGCAAGAAGAG
AAGGCCAAGCTGGAAG

Lonicera japonica flavone synthase (*FNS1*) mRNA, partial cds **(JX068612.1)**

TTTTCACCCTACGGTCCCTATTGGAAATTCATCAAAAAAACATGCACCTTTGAACTTTTGGGCACACGTAACATG
AACCACTTTCTCCCCATTAGGACCAACGAGATTCGTCGTTTCTTACAAGTGATGTTAGAAAAAGCCAAGGCTAGT
GAGGGGGTGAACGTGACTGAAGAGTTGATCAAGCTCACGAACAACGTTATCTCTCAAATGATGTTTAGTACTCGG
AGCTCGGGGACCGAGGGGGAGGCGGAGGAGATGAGGACATTGGTACGTGAGGTGACTCAAATATTCGGAGAATTT
AATGTTTCGGATTTTATAAAGTTGTGTAAGAACATTGATATTGGAGGGTTTAAGAAGAGAAGT

Dahlia pinnata *DvFNS1* mRNA for flavone synthase, complete cds **(AB769842.1)**

AATCTCATCTTACCATGAATACACTCCTAGTACTCCAAATGGTAATTCCGGCAATAATTGCCTTTGTAAT
CTTCCACTTATTATTCTTCAAAAGCAAACCGAACCGTCGTCTCCCACCCAGCCCCCCATCATTACCAATT
ATTGGCCACCTCCACCACCTCGGCCCGCTCATCCACCAATCCTTCAATCGCCTATCCGCCCGCTACGGTC
CACTAATCCACCTCCGTCTCGGTTCGGTCTCATGTGTCGTCGCAGACGCTCCTGACCTCGCCCAAGAATT
ACTCCAAAAAAACGACCTCGCCTTCGCTAATCGAAAACACACCTTAGCCATTGACCATGTCACGTACGGT
GTCGCCTTTGCCTTCGCACCTTACGGTCCTTATTGGAGATTTATTAAAAAAATGTCCACGGTCGAGCTTT
TGGGCATCCAGAATCTCGGCCATTTCCTCCCCATTCGAACCCAAGAAATCCACGGACTTCTACTAACATT
AACGGAGAAATCCAAACAAAATGAGAGTGTGAACATGACGAATGAGTTGTTAAAGCTATCAAACAACATT
ATCTGTCAAATGATGATGGGTATCCGATGTTCCGGGAACAAAACTGAAGCAGAAGAAGCGAAAAACCTAG
TGCGAGAAGTGACAACGATATTCGGAGAGTTTAATGTTTCGGATTTTATATGGTTTTGCAAGAAGTTAGA
TTTGCAAGGGTTTAAGAAGAGGTACGAGGATATACGTACAAGGTATGATGCTTTACTTGAAAGGATAATA
TTTGCAAGGGAAGAGATGAGAAAAGAAGGGAAGGGTATGGAGGATGGCAAAGGGAAAGATTTTCTAGATA
TGTTGCTTGATGTTTTGGAGGATGACAAAGCTGAGATTAAGATTACCAGAAACCATATCAAAGCCTTGAT
TTTGGACTTCGTTACAGCTGGAACAGACACAACCGCAGTAATAATAGAATGGACATTAGTAGAACTTATC
AAGAACCCTATGGTCATGGAAAAAGCAAAACAAGAGCTCGATGAAGTCGTCGGAAACACGAGGTTAGTCG
AGGAGTCGGATGCACCGAAGCTGCCTTACATTCAAGCAATCATAAAAGAAGCCTTCCGACTTCATCCACC
AATTCCAATGATTATACGGAAGTCAAATGAAAATGTGAGTGTTAAATCCGGATATGAAATCCCCGCAGGG
TCCATATTGTTTGTTAACAATTGGTCAATTGGACGAAACCCAAAATACTGGGAAAGCCCATTAGAGTTCA
AGCCAGATAGGTTTTTAAAAGAGGGTGTCCTTAAACCCTCGTTAGATATTAGGGGCCAAAACTTTCAAAT
TTTGCCTTTTGGAACCGGGAGGAGAAGTTGTCCTGGTATTAATATGGCTATGAGACAACTCCCTGTGGTG
GTTGCCATCCTCATACAATGCTTTGAATGGACGGTTAATGATAAACAAGTGTTGAATATGGATGAACGAG
GTGGACTCACCACTCCAAGGGCAACAGATCTAGTGTGTTTCCCTTTGCTTCGTAAAAACTCTCCACATTC
GATGTTTACTTCAGTCTGAGTGTTGTTTAATTTATGATGGATATTACCGTTGGTGATAGTCCTTTATCTA
ATAACGAATCTAGTGTGTTTCCCTTCGCTTCGC

Fig. S5 (continued) Partial sequence of flavone synthase (*FNS*) gene in the studied plant species of Apiaceae and *Lonicera japonica* and *Dahlia pinnata* as an outgroup species.

Table S3: The nucleotide composition of partial sequence flavone synthase (*FNS*) gene in the studied plant species of Apiaceae.

Plant species	Flavone content	length (bp)	Σ GC%	Σ AT%	G	C	A	T
D. carota	Low flavone content	569	47.80	52.20	20.74	27.07	23.55	28.65
P. anisum		501	44.11	55.89	26.55	17.56	31.34	24.55
E. campestre		497	45.07	54.93	26.36	18.71	30.18	24.75
C. carvi		491	43.99	56.01	26.48	17.52	30.96	25.05
T. arvensis		642	43.30	56.70	25.23	18.07	31.78	24.92
D. tortuosa	High flavone content	681	41.85	58.15	23.94	17.91	29.81	28.34
Ap. graveolens		594	43.43	56.57	24.58	18.86	29.29	27.27
C. cyminum		501	43.91	56.09	26.35	17.56	31.34	24.75
P. crispum		492	44.51	55.49	26.22	18.29	30.28	25.20
**Total average**		552	44.22	55.78	25.04	19.10	29.79	26.07

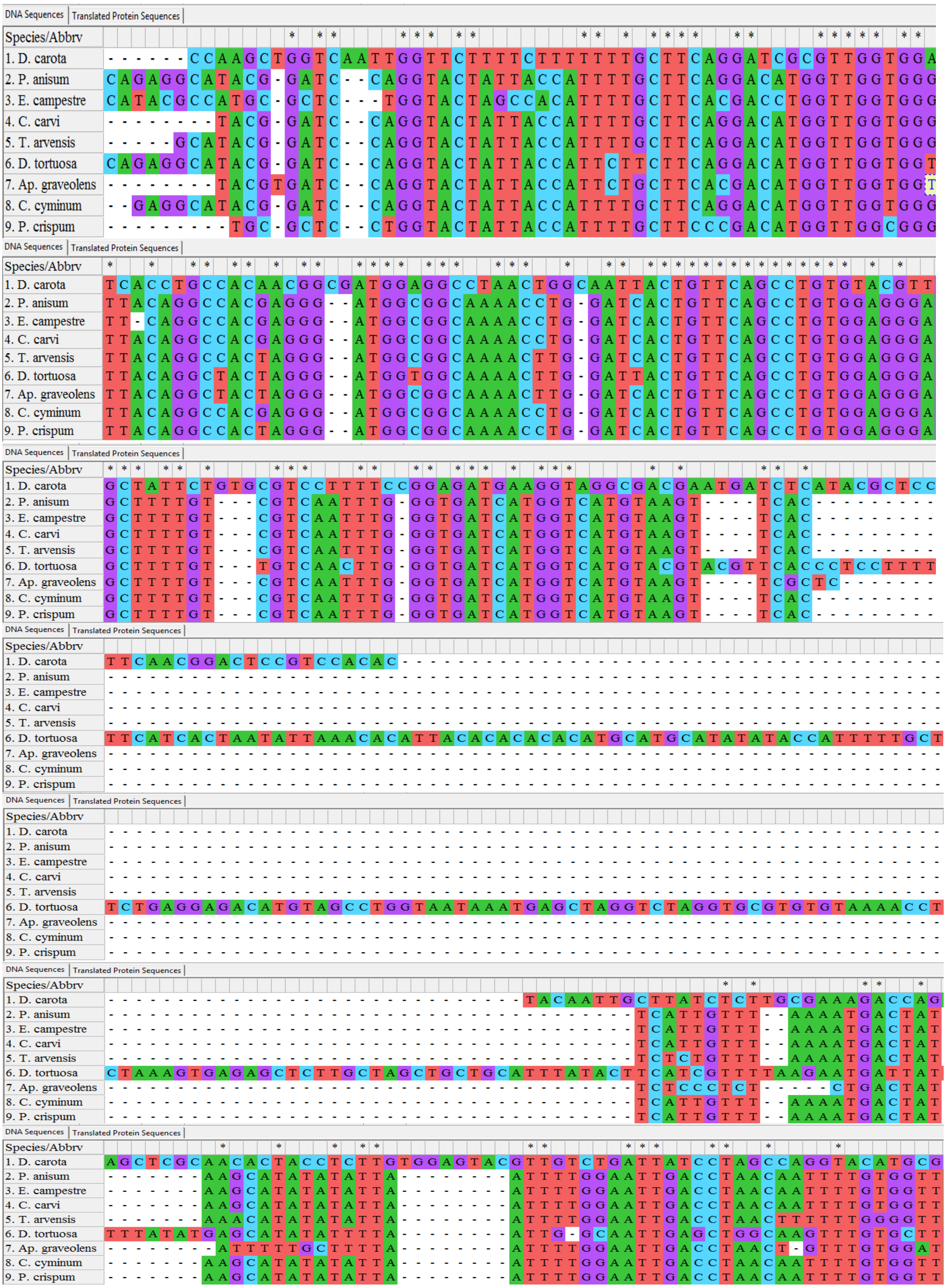

Fig. S6 Alignment of partial DNA sequences of flavone synthase (*FNS*) gene isolated from the studied plant species by MEGA-X.

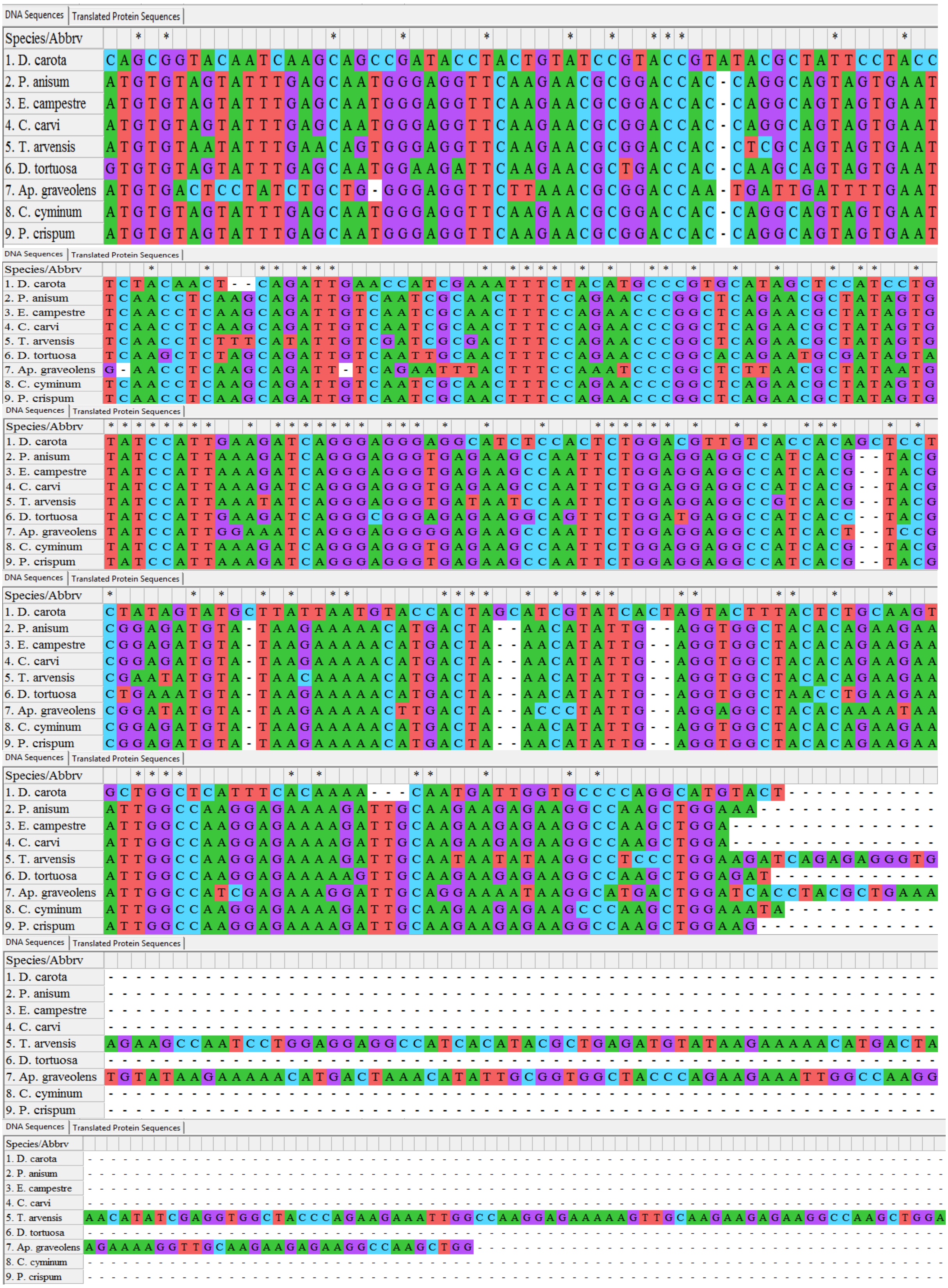

Fig. S6 (continued) Alignment of partial DNA sequences of flavone synthase (*FNS*) gene isolated from the studied plant species by MEGA-X.
